# KCNJ2 is Required for NLRP3 Inflammasome Activation That Drives Allergic Airway Inflammation and Remodeling

**DOI:** 10.1002/advs.202517666

**Published:** 2026-04-10

**Authors:** Yachao Cui, Shumei Wu, Yang Peng, Yiqi Liu, Li Che, Shiying Chen, Feng Zhang, Dajiang Qin, Shiyue Li, Pixin Ran, Wenguang Yin

**Affiliations:** ^1^ State Key Laboratory of Respiratory Disease National Clinical Research Center for Respiratory Disease National Center for Respiratory Medicine Joint International Research Laboratory of Respiratory Health Guangdong Basic Research Center of Excellence for Respiratory Medicine Guangzhou Institute of Respiratory Health Guangzhou National Laboratory China‐Portugal Artificial Intelligence and Public Health Technologies Joint Laboratory the First Affiliated Hospital of Guangzhou Medical University Guangzhou P. R. China; ^2^ Guangzhou National Laboratory Guangzhou International Bio Island Guangzhou Guangdong P. R. China; ^3^ China‐Portugal Artificial Intelligence and Public Health Technologies Joint Laboratory Guangdong‐Hong Kong‐Macao Joint Laboratory of Respiratory Infectious Diseases Guangdong Provincial Key Laboratory of Respiratory Disease Research Guangzhou Medical University Guangzhou Guangdong P. R. China; ^4^ Key Laboratory of Biological Targeting Diagnosis Therapy and Rehabilitation of Guangdong Higher Education Institutes the Fifth Affiliated Hospital of Guangzhou Medical University Guangzhou Guangdong P. R. China

**Keywords:** asthma, airway inflammation, airway remodeling, KCNJ2, NLRP3 inflammasome

## Abstract

Airway inflammation and remodeling are cardinal features of asthma pathogenesis. Genome‐wide association studies have shown that several SNPs of *KCNJ2*, a member of the inwardly rectifying potassium channel family, are associated with asthma in patients. However, the role of KCNJ2 in airway inflammation and remodeling in asthma remains unknown. Here, we demonstrate that the Kcnj2 serves as a critical regulator of airway epithelial inflammation and remodeling. KCNJ2 expression is significantly reduced in the airway epithelium of asthmatic patients, which is associated with goblet cell metaplasia and mucus overproduction. Epithelial cell depletion of *Kcnj2* attenuates airway inflammation, Th2 inflammatory response, goblet cell metaplasia, and mucus overproduction in the airways of asthmatic mice. In cultured primary airway epithelial cells of asthmatic patients, KCNJ2 inhibition also hampers goblet cell metaplasia, mucus production, and lung epithelial cell‐derived alarmins expression. This process appears to be mediated, at least in part, through inhibition of NLRP3 by restricting Ca^2+^ influx and K^+^ efflux, as pharmacological activation of NLRP3 diminishes the KCNJ2 inhibition‐ameliorated airway phenotypes. These results provide insight into the role of Kcnj2 in airway inflammation and remodeling in asthmatic conditions.

## Graphical abstract

This study aims to evaluate the impact of the potassium channel KCNJ2 on asthma development. KCNJ2 promotes NLRP3 inflammasome activation through both Ca^2^^+^ influx and K^+^ efflux in airway epithelial cells, which drives allergic airway inflammation and remodeling, suggesting a promising therapeutic target for asthma.

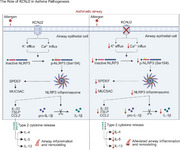

## 1 Introduction

Asthma is a chronic airway disease affecting an estimated 262.4 million people worldwide in 2019, imposing a substantial burden on healthcare systems and economies [1]. Asthma triggers include allergens (pollens, molds, dust, and pet dander), tobacco smoke, exercise, air pollutants, weather patterns and/or changes, and virus infections [2]. Although the precise causes of asthma are unclear, strong genetic predisposition and environmental drivers of asthma contribute to the onset of the disease [3]. Airway remodeling is one of the common pathological changes in asthma, and includes goblet cell hyperplasia and mucus overproduction, important causes of morbidity and mortality [4, 5]. In animal models of asthma, goblet cell hyperplasia has been shown to be regulated by transcription factors including SPDEF, RUNX2, FOXM1, FOXA2, and NKX2‐1 [6, 7, 8, 9, 10, 11], as well as by their upstream regulators, including RCM‐1, a FOXM1 inhibitor, in airway epithelial cells [12]. However, it remains unclear how these transcription factors or regulators, such as Spdef in goblet cell differentiation are regulated.

Airway inflammation is predominantly driven by type 2 T helper (Th2) cells and group 2 innate lymphoid cells (ILC2s) for many asthmatic cases [13]. The Th2 cell cytokines, including interleukin (IL)‐4, IL‐5, and IL‐13, orchestrate hallmark features of asthma including airway inflammation, airway remodeling, airway hyperresponsiveness (AHR), and mucus overproduction [5, 14]. For example, goblet cell hyperplasia and mucin production can be driven by Th2 cytokines such as IL‐4 and IL‐13 in asthma [5, 14]. IL‐13 promotes goblet cell differentiation by inducing SPDEF expression in asthmatic mouse models [8]. However, the upstream regulation of Th2 inflammation, including Th2 cytokines expression during asthma pathogenesis, remains to be fully elucidated.

Inwardly rectifying (Kir) channels such as Kir2.1 (encoded by *Kcnj2*) and Kir7.1 (encoded by *Kcnj13*) are recently reported to mediate face and trachea development in mice [15, 16, 17]. Genome‐wide association studies (GWAS) show that changes in pulmonary function are associated with single‐nucleotide polymorphisms (SNPs) in potassium channel genes *KCNJ2* and *KCNK1* [18, 19]. A more recent GWAS study reveals that severe asthma exacerbation is associated with a novel SNP that is associated with expression of *KCNJ2* antisense RNA 1 [20]. Despite these associations, the role for *Kcnj2* in pulmonary diseases, especially in asthma, remains unknown.

NLRP3 inflammasome is a multiprotein complex composed of three interacting proteins (NLRP3, ASC, and procaspase‐1) [21, 22]. Besides regulating inflammation in autoinflammatory diseases such as cryopyrin‐associated periodic syndrome in humans, NLRP3 has also been shown to be associated with or may contribute to a range of chronic inflammatory diseases revealed by clinical and/or animal disease model studies [23]. Studies report that NLRP3 inflammasome is upregulated in asthmatic patients [24, 25]. Although NLRP3 inhibition can relieve airway inflammation in animal models of asthma [26, 27, 28], it remains unknown how NLRP3 inflammasome activation is regulated in this process. In addition, global inactivation of NLRP3 has been reported to inhibit airway inflammation together with goblet cell hyperplasia in animal models of asthma [26]. NLRP3 can also maintain the expression of SPDEF, a key regulator of MUC5AC expression in S. pneumoniae‐infected mice or in hRV16‐infected human nasal epithelial cells [29, 30]. However, the role of NLRP3 in airway epithelial cells in asthma development and how its activation is regulated in this process remain unknown.

Decreased intracellular K^+^ or elevated intracellular Ca2^+^ levels are known to promote NLRP3 activation [31, 32, 33, 34, 35, 36]. Several potassium channels, including *Kcnk6* (encoding TWIK2) and *Kcnk13* (encoding THIK‐1), have been reported to trigger NLRP3 activation in macrophages by facilitating K^+^ efflux [32, 33, 34]. KCNJ2 has been reported to facilitate both K^+^ efflux and Ca2^+^ influx [37, 38, 39]. One study also shows that KCNJ2 plays an important role in driving inflammation triggered by pathogenic signals [40]. For example, pharmacologic inhibition of KCNJ2 using ML133 or myeloid cell‐specific deletion (*Lyz2‐cre‐Kcnj2^f/f^*) reduces serum levels of IL‐1β, IL‐1α, and IL‐6 in an in vivo LPS‐induced sepsis model [40]. Whether and how KCNJ2 regulates NLRP3 activation, particularly in lung diseases including asthma, remains unknown.

Here, we investigate the function of KCNJ2 in allergic asthma. We reveal a role for KCNJ2 in the regulation of airway inflammation and remodeling in asthma, at least in part via the control of NLRP3‐mediated Th2 inflammation and goblet cell differentiation.

## 2 Results

### 2.1 KCNJ2 Expression is Downregulated in the Airway Epithelium of Asthmatic Patients

**FIGURE 1:**
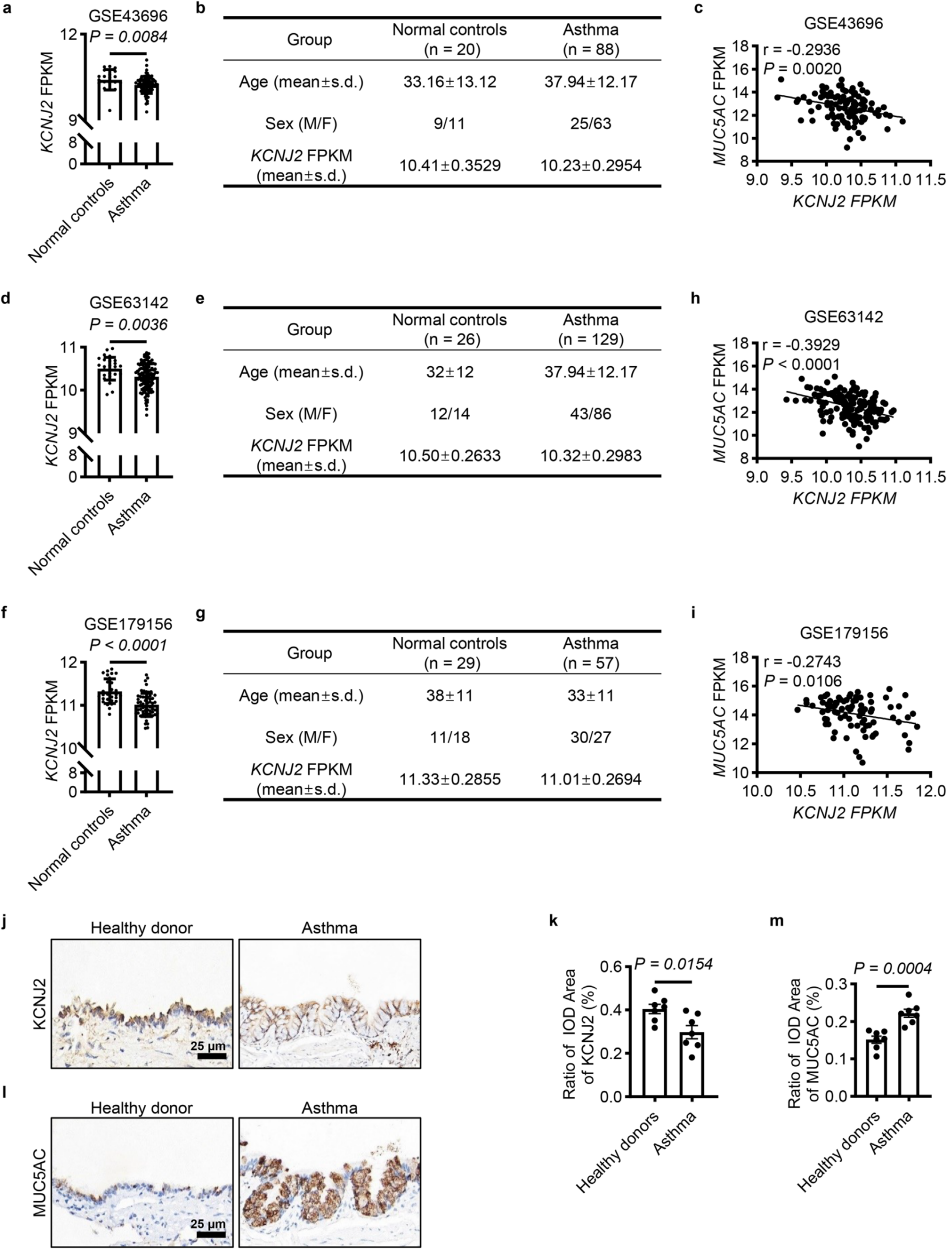
KCNJ2 expression is reduced in the airway epithelium of asthmatic patients. (a) Quantification of *KCNJ2* mRNA levels in primary bronchial epithelial cells in normal control (*n* = 20) and asthmatic patients (*n* = 88) in GSE43696 (microarray: Agilent‐014850 Whole Human Genome Microarray 4×44K G4112F). (b) Information of normal controls and asthmatic patients in GSE43696. (c) Correlations between *KCNJ2* mRNA levels and *MUC5AC* mRNA levels in primary bronchial epithelial cells of normal controls and asthmatic patients in GSE43696. (d) Quantification of *KCNJ2* mRNA levels in primary bronchial epithelial cells in normal controls (*n* = 26) and asthmatic patients (*n* = 129) in GSE63142 (microarray: Agilent‐014850 Whole Human Genome Microarray 4×44K G4112F). (e) Information of normal controls and asthmatic patients in GSE63142. (f) Quantification of *KCNJ2* mRNA levels in primary bronchial epithelial cells in normal controls (*n* = 29) and asthmatic patients (*n* = 57) in GSE179156 (microarray: HG‐U133_Plus_2 Affymetrix Human Genome U133 Plus 2.0 Array). (g) Information of normal control and asthmatic patients in GSE179156. (h) Correlations between *KCNJ2* mRNA levels and *MUC5AC* mRNA levels in primary bronchial epithelial cells of normal controls and asthmatic patients in GSE63142. (i) Correlations between *KCNJ2* mRNA levels and *MUC5AC* mRNA levels in primary bronchial epithelial cells of normal controls and asthmatic patients in GSE179156. (j) Representative images of immunohistochemistry for KCNJ2 in healthy donors (*n* = 6) and asthmatic patients (*n* = 6). (k) Quantification of average optical density values of KCNJ2 (as in j). (l) Representative images of immunohistochemistry for MUC5AC in healthy donors (*n* = 6) and asthmatic patients (*n* = 6). (m) Quantification of average optical density values of MUC5AC (as in l). Data are shown as mean ± s.d. Mann‐Whitney test (a, d, f), Unpaired Students’ *t*‐test (k, m), Pearson correlation coefficient (c, h, i).

To explore a potential role for *KCNJ2* in asthma, we first assessed changes in mRNA levels of *KCNJ2* in bronchial epithelial cells obtained from asthmatic patients by bronchoscopy and epithelial brushing by analyzing a microarray dataset of GSE43696 (Agilent‐014850 Whole Human Genome Microarray 4×44K G4112F) containing 20 normal controls and 88 patients with asthma. *KCNJ2* levels were decreased in bronchial epithelial cells of asthmatic patients compared with normal controls (Figure 1a,b). Interestingly, *KCNJ2* expression showed a negative correlation with that of *MUC5AC* (Figure 1c). Consistent findings were observed in another two microarray datasets, GSE63142 (26 normal controls and 129 patients with asthma) and GSE179156 (29 normal controls and 57 patients with asthma). In both cohorts, *KCNJ2* levels were decreased in bronchial epithelial cells of asthmatic patients compared with their corresponding normal controls (Figure 1d–g). Moreover, *KCNJ2* expression also exhibited a negative correlation with that of *MUC5AC* (Figure 1h,i). Next, we examined KCNJ2 expression in the lungs of healthy donors and asthmatic patients by immunohistochemistry (Table S1). KCNJ2 was also expressed in airway epithelial cells (Figure 1j). We found that asthmatic patients exhibited reduced KCNJ2 levels in the airway epithelium compared with healthy donors (Figure 1j,k), accompanied by increased MUC5AC levels and goblet cell hyperplasia (Figure 1l,m). These results suggest that a decrease in KCNJ2 is associated with, and may contribute to, airway remodeling, including goblet cell hyperplasia and mucus overproduction in asthmatics.

### 2.2 *Kcnj2* Deficiency in Lung Epithelial Cells Attenuates Airway Hyperresponsiveness, Airway Inflammation, Th2 Immune Responses, and Expression of Alarmins in OVA‐Induced Asthmatic Mice

**FIGURE 2:**
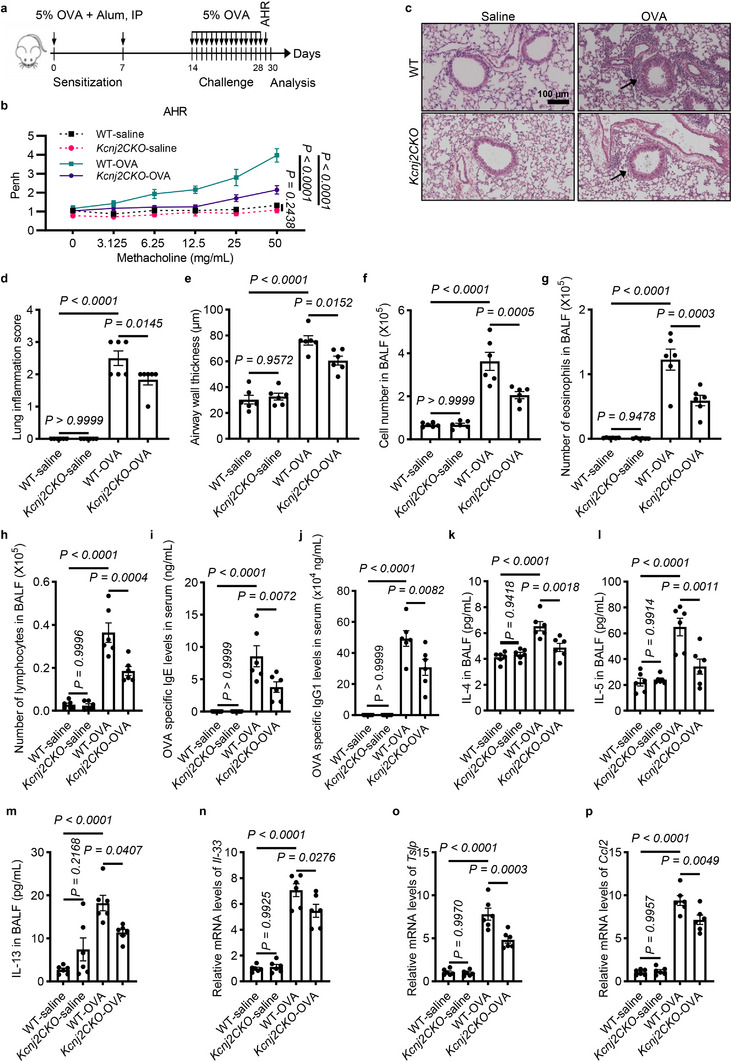
Lung epithelial cell deletion of *Kcnj2* inhibits airway hyperresponsiveness, airway inflammation and Th2 immune responses in OVA‐induced asthmatic mice. (a) Timeline for the OVA‐induced asthmatic mouse model, indicating time points for sensitization, challenge, and analysis (IP: Intraperitoneal). Sensitization: 100 µL 5% OVA + 100 µL Alum; Challenge: 5% OVA, 30 min. (b) Penh measurements in response to serially increasing doses of aerosolized methacholine in WT‐saline (*n* = 6) and WT‐OVA (*n* = 6) mice, and *Kcnj2CKO*‐saline (*n* = 6) and *Kcnj2CKO*‐OVA (*n* = 6) mice. (c) Representative images of lung tissue sections stained with hematoxylin and eosin from WT‐saline (*n* = 6) and WT‐OVA (*n* = 6) mice, and *Kcnj2CKO*‐saline (*n* = 6) and *Kcnj2CKO*‐OVA (*n* = 6) mice. Arrows point to airway inflammatory cells. (d) Quantification of inflammation score (as in c). (e) Quantification of airway wall thickness (as in c). Numbers of total cells (f), eosinophils (g), and lymphocytes (h) in BALF of WT‐saline (*n* = 6) and WT‐OVA (*n* = 6) mice, and *Kcnj2CKO*‐saline (*n* = 6) and *Kcnj2CKO*‐OVA (*n* = 6) mice. (i) OVA‐specific IgE levels in the serum of WT‐saline (*n* = 6) and WT‐OVA (*n* = 6) mice, and *Kcnj2CKO*‐saline (*n* = 6) and *Kcnj2CKO*‐OVA (*n* = 6) mice. (j) OVA‐specific IgG1 levels in the serum of WT‐saline (*n* = 6) and WT‐OVA (*n* = 6) mice, and *Kcnj2CKO*‐saline (*n* = 6) and *Kcnj2CKO*‐OVA (*n* = 6) mice. Protein levels of IL‐4 (k), IL‐5 (l), and IL‐13 (m) in BALF of WT‐saline (*n* = 6) and WT‐OVA (*n* = 6) mice, and *Kcnj2CKO*‐saline (*n* = 6) and *Kcnj2CKO*‐OVA (*n* = 6) mice by ELISA. RT‐qPCR analysis of *Il‐33* (n), *Tslp* (o), and *Ccl2* (p) in the lungs of WT‐saline (*n* = 6) and WT‐OVA (*n* = 6) mice, and *Kcnj2CKO*‐saline (*n* = 6) and *Kcnj2CKO*‐OVA (*n* = 6) mice. Data are shown as mean ± s.d., two‐way ANOVA (b), one‐way ANOVA (d–p). OVA, ovalbumin; Alum, aluminum hydroxide; WT, wild‐type; BALF, bronchoalveolar lavage fluid.

To investigate the role of *Kcnj2* in asthma development in vivo, we used OVA‐sensitized and OVA‐challenged mice as animal models of allergic asthma (Figure 2a). Because *Kcnj2* is highly expressed in the lung epithelium, we inactivated *Kcnj2* specifically in lung epithelial cells using the *Nkx2.1^Cre^* line that can efficiently delete genes in the lung epithelium, including the airway epithelium [41]. As expected, *Nkx2.1^Cre^; Kcnj2^flox/flox^* (*Kcnj2CKO*) mice exhibited dramatically reduced levels of *Kcnj2* mRNA and proteins in the lungs, including the airway epithelium (Figure S1a–c). Airway hyperresponsiveness (AHR) is a hallmark feature of asthma. We used the enhanced pause (Penh) as an index of airway responsiveness to increased doses of aerosolized methacholine in asthmatic mice (Figure 2b). *Kcnj2CKO‐*saline mice exhibited no obvious differences in AHR to methacholine compared with WT‐saline mice (Figure 2b). As expected, OVA sensitization and challenge led to increased AHR to methacholine in WT mice compared with the saline group (Figure 2b). Notably, *Kcnj2CKO*‐OVA mice displayed attenuated AHR compared with WT‐OVA mice (Figure 2b). Next, we evaluated the effect of *Kcnj2* on OVA‐induced lung inflammation. *Kcnj2CKO*‐saline mice displayed no obvious inflammation in the lungs, similar to WT‐saline mice (Figure 2c,d). *Kcnj2CKO*‐OVA mice exhibited reduced inflammatory cell infiltration in peribronchial and perivascular regions compared with WT‐OVA mice (Figure 2c,d). Consistently, *Kcnj2CKO*‐saline mice displayed no obvious differences in numbers of total cells, eosinophils, or lymphocytes in bronchoalveolar lavage fluid (BALF) compared with WT‐saline mice (Figure 2f–h). Interestingly, *Kcnj2CKO*‐OVA mice exhibited decreased numbers of total cells, eosinophils, and lymphocytes in the BALF compared with WT‐OVA mice (Figure 2f–h). OVA sensitization and challenge also increased serum immunoglobulin E (IgE) and immunoglobulin G1 (IgG1) levels in WT‐OVA mice compared with WT‐saline mice (Figure 2i,j). These elevations were attenuated in *Kcnj2CKO*‐OVA mice (Figure 2i,j). Allergen exposure can cause allergen‐specific Th2 cell activation and production of Th2 cytokines, such as IL‐4, IL‐5, and IL‐13, which promote airway inflammation, airway remodeling, AHR, and mucus overproduction [5, 13, 42]. We subsequently examined the effect of *Kcnj2* deficiency on the production of IL‐4, IL‐5, and IL‐13. OVA sensitization and challenge increased levels of IL‐4, IL‐5, and IL‐13 in the BALF of WT mice compared with WT‐saline mice (Figure 2k–m). *Kcnj2CKO*‐OVA mice displayed decreased levels of IL‐4, IL‐5, and IL‐13 in the BALF compared with those of WT‐OVA mice (Figure 2k–m). Collectively, these findings suggest that epithelial KCNJ2 functions to promote Th2 inflammation during asthma development.

Alarmin cytokines can be rapidly produced in response to allergen insults in the lungs. Lung epithelial cell‐derived alarmins, including IL‐33, TSLP, and CCL2, play critical roles in orchestrating Th2 immune responses in allergic lung inflammation [43, 44]. We hypothesized that KCNJ2 deficiency may suppress the production of these alarmins. As expected, OVA sensitization and challenge increased levels of *Il‐33*, *Tslp*, and *Ccl2* in the lungs compared with the saline group (Figures 2n‐p). Importantly, levels of *Il‐33*, *Tslp*, and *Ccl2* were significantly reduced in the lungs of *Kcnj2*‐OVA mice compared with those of WT‐OVA mice (Figure 2n–p). However, we found no obvious differences in levels of *Il‐33*, *Tslp*, or *Ccl2* in the lungs between *Kcnj2CKO*‐saline mice and WT‐saline mice (Figure 2n–p). Together, these data indicate that KCNJ2 inactivation attenuates Th2 inflammation by suppressing the production of lung epithelial cell‐derived alarmins, thereby alleviating airway inflammation in asthmatic conditions.

### 2.3 *Kcnj2* Deficiency in Lung Epithelial Cells Attenuates Goblet Cell Hyperplasia and Mucus Overproduction in OVA‐Induced Asthmatic Mice

**FIGURE 3:**
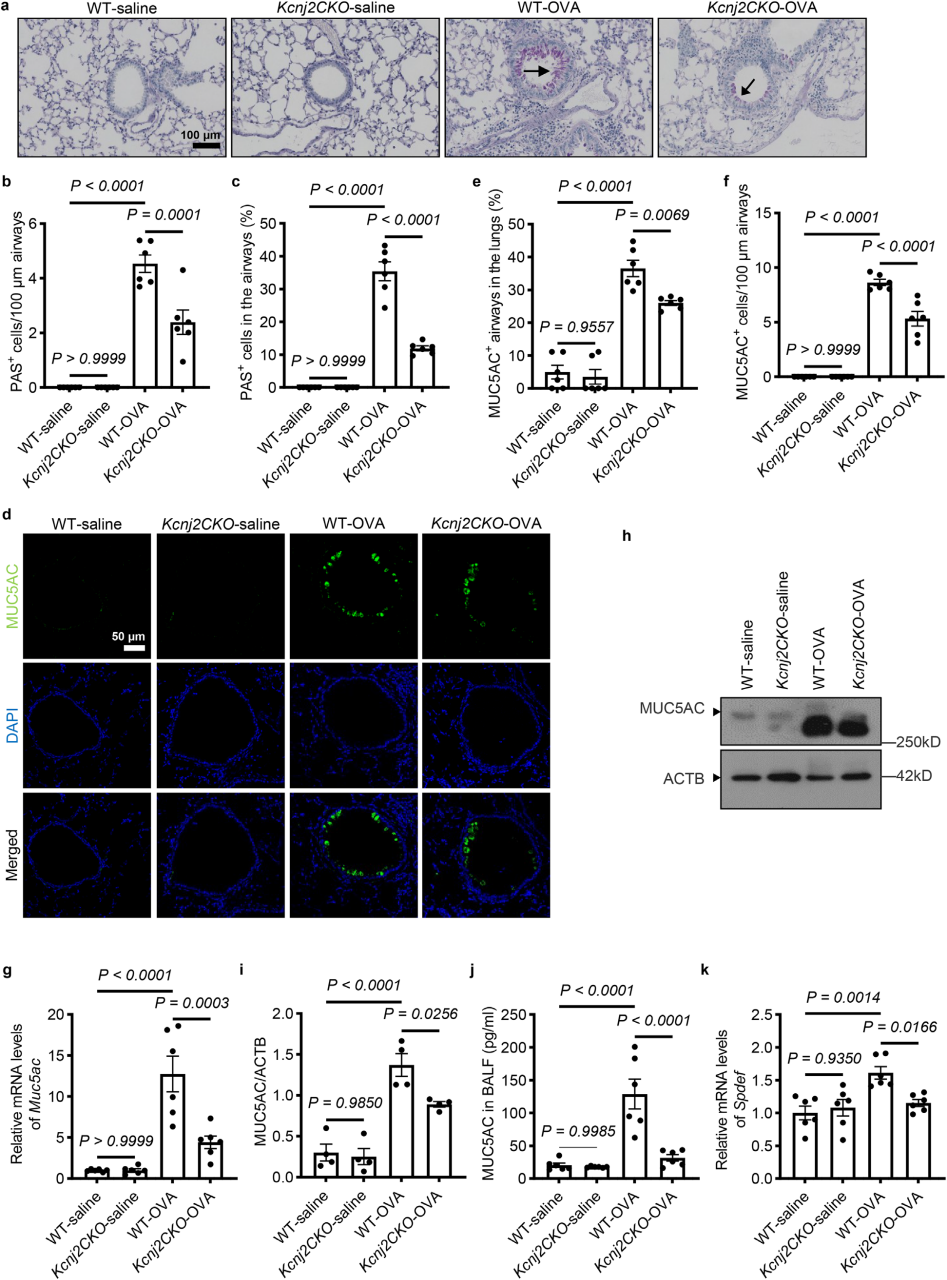
Epithelial cell deletion of *Kcnj2* inhibits airway goblet cell metaplasia and mucus overproduction in OVA‐induced asthmatic mice. (a) Representative images of lung tissue sections stained with Periodic acid‐Schiff (PAS) of WT‐saline (*n* = 6) and WT‐OVA (*n* = 6) mice, and *Kcnj2CKO*‐saline (*n* = 6) and *Kcnj2CKO*‐OVA (*n* = 6) mice. Arrows point to PAS^+^ goblet cells. (b) Quantification of PAS^+^ cells per 100‐µm airway of WT‐saline (*n* = 6) and WT‐OVA (*n* = 6) mice, and *Kcnj2CKO*‐saline (*n* = 6) and *Kcnj2CKO*‐OVA (*n* = 6) mice. 2, 0, 2721 and 1435 PAS ^+^ cells were analyzed for WT‐saline (*n* = 6) and WT‐OVA (*n* = 6) mice, and *Kcnj2CKO*‐saline (*n* = 6) and *Kcnj2CKO*‐OVA (n = 6) mice, respectively. (c) Percentage of PAS^+^ cells in the airway epithelium of WT‐saline (n = 6) and WT‐OVA (n = 6) mice, and *Kcnj2CKO*‐saline (n = 6) and *Kcnj2CKO*‐OVA (*n* = 6) mice. d Immunostaining for MUC5AC (green) and DAPI staining (blue) of lung sections of WT‐saline (*n* = 6) and WT‐OVA (*n* = 6) mice, and *Kcnj2CKO*‐saline (*n* = 6) and *Kcnj2CKO*‐OVA (*n* = 6) mice. (e) Percentage of airways with MUC5AC^+^ goblet cells in the lungs of WT‐saline (*n* = 6) and WT‐OVA (*n* = 6) mice, and *Kcnj2CKO*‐saline (*n* = 6) and *Kcnj2CKO*‐OVA (*n* = 6) mice. (f) Quantification of MUC5AC^+^ goblet cells per 100‐µm airway of WT‐saline (*n* = 6) and WT‐OVA (*n* = 6) mice, and *Kcnj2CKO*‐saline (*n* = 6) and *Kcnj2CKO*‐OVA (*n* = 6) mice. 36, 45, 4217 and 2881 MUC5AC^+^ goblet cells were analyzed for WT‐saline (*n* = 6) and WT‐OVA (*n* = 6) mice, and *Kcnj2CKO*‐saline (*n* = 6) and *Kcnj2CKO*‐OVA (*n* = 6) mice, respectively. (g) RT‐qPCR analysis of *Muc5ac* in the lungs of WT‐saline (*n* = 6) and WT‐OVA (*n* = 6) mice, and *Kcnj2CKO*‐saline (*n* = 6) and *Kcnj2CKO*‐OVA (*n* = 6) mice. (h) Western blotting for MUC5AC and ACTB in lung tissues of WT‐saline (*n* = 4) and WT‐OVA (*n* = 4) mice, and *Kcnj2CKO*‐saline (*n* = 4) and *Kcnj2CKO*‐OVA (*n* = 4) mice. (i) Quantification of relative MUC5AC levels (as in h). (j) Protein levels of MUC5AC in BALF of WT‐saline (*n* = 6) and WT‐OVA (*n* = 6) mice, and *Kcnj2CKO*‐saline (*n* = 6) and *Kcnj2CKO*‐OVA (*n* = 6) mice by ELISA. (k) RT‐qPCR analysis of *Spdef* in the lungs of WT‐saline (*n* = 6) and WT‐OVA (*n* = 6) mice, and *Kcnj2CKO*‐saline (*n* = 6) and *Kcnj2CKO*‐OVA (*n* = 6) mice. Data are shown as mean ± s.d., one‐way ANOVA.

Goblet cell hyperplasia and mucus overproduction are hallmark features of asthma. Next, we examined the effect of KCNJ2 on goblet cell differentiation in the lungs by Periodic Acid‐Schiff (PAS) staining and immunofluorescence staining. We observed that there were rare PAS^+^ (Figure 3a–c) or MUC5AC^+^ (Figure 3d–f) goblet cells in the airways of WT‐saline mice. OVA sensitization and challenge induced hyperplasia of both PAS^+^ (Figure 3a–c) and MUC5AC^+^ (Figure 3d–f) goblet cells in the lungs of WT mice compared with WT‐saline mice. Interestingly, *Kcnj2CKO*‐OVA mice exhibited reduced numbers of PAS^+^ (Figure 3a–c) and MUC5AC^+^ (Figure 3d–f) goblet cells in the airways compared with those of WT‐OVA mice. However, we found no obvious differences in the number of PAS^+^ (Figure 3a–c) or MUC5AC^+^ (Figure 3d–f) goblet cells between *Kcnj2CKO*‐saline mice and WT‐saline mice. OVA‐immunized C57BL/6 mice have been reported to show increased airway wall thickness, possibly due to goblet cell hyperplasia and airway smooth muscle hyperplasia and/or hypertrophy [45]. Consistently, WT‐OVA mice exhibited increased airway wall thickness compared with WT‐saline mice (Figure 2e). Importantly, *Kcnj2CKO*‐OVA mice displayed reduced airway wall thickness compared with WT‐OVA mice (Figure 2e). However, we found no obvious changes in airway wall thickness between *Kcnj2CKO*‐saline mice and WT‐saline mice (Figure 2e). OVA sensitization and challenge also induced increased mRNA levels of *Muc5AC* and overproduction of MUC5AC, a major mucin expressed and secreted by goblet cells in the lungs (Figure 3g–i). mRNA levels of *Muc5AC* were reduced in the lungs of *Kcnj2CKO*‐OVA mice compared with WT‐OVA mice, but not between *Kcnj2CKO*‐saline mice and WT‐saline mice (Figure 3g). Consistent with these findings, MUC5AC protein levels in lung tissues (Figure 3h,i) and in the BALF (Figure 3j) were decreased in *Kcnj2CKO*‐OVA mice compared with those of WT‐OVA mice, but not between *Kcnj2CKO*‐saline mice and WT‐saline mice (Figure 3h,i). However, OVA sensitization and challenge caused no significant changes in the number of MUC5B^+^ secretory cells or MUC5B production (Figure S2a–c), consistent with previous reports [46]. *Kcnj2CKO*‐saline mice exhibited no obvious differences in the number of MUC5B^+^ cells in the airways (Figure S2a,b) or MUC5B protein levels in the BALF (Figure S2c) compared with WT‐saline mice. In addition, we observed no obvious changes in the number of MUC5B^+^ cells in the airways (Figure S2a,b) or MUC5B protein levels in the BALF (Figure S2c) between *Kcnj2CKO*‐OVA and WT‐OVA mice. These results indicate that KCNJ2 is required for goblet cell hyperplasia, which may lead to MUC5AC^+^ mucus overproduction and hypersecretion in asthmatic conditions.

To determine how KCNJ2 regulates goblet cell differentiation in asthmatic conditions, we examined changes in expression of *Spdef* and *Foxa3*, two key transcription factors known to promote goblet cell differentiation and hyperplasia [6, 8]. OVA sensitization and challenge led to increased mRNA levels of *Spdef* and *Foxa3* in the lungs of WT mice compared with WT‐saline mice (Figure 3k and Figure S3). Interestingly, *Spdef* levels in the lungs were reduced in *Kcnj2CKO*‐OVA mice compared with WT‐OVA mice, but not between *Kcnj2CKO*‐saline mice and WT‐saline mice (Figure 3k). However, we observed no significant differences in *Foxa3* levels between *Kcnj2CKO*‐OVA mice and WT‐OVA mice (Figure S3), or between *Kcnj2CKO*‐saline mice and WT‐saline mice (Figure S3). Altogether, these data suggest that *Kcnj2* promotes goblet cell hyperplasia and MUC5AC^+^ mucus overproduction by regulating *Spdef* expression during asthma development.

### 2.4 *Kcnj2* Deficiency in Lung Epithelial Cells Inhibits NLRP3 Inflammasome Activation in OVA‐Induced Asthmatic Mice

**FIGURE 4:**
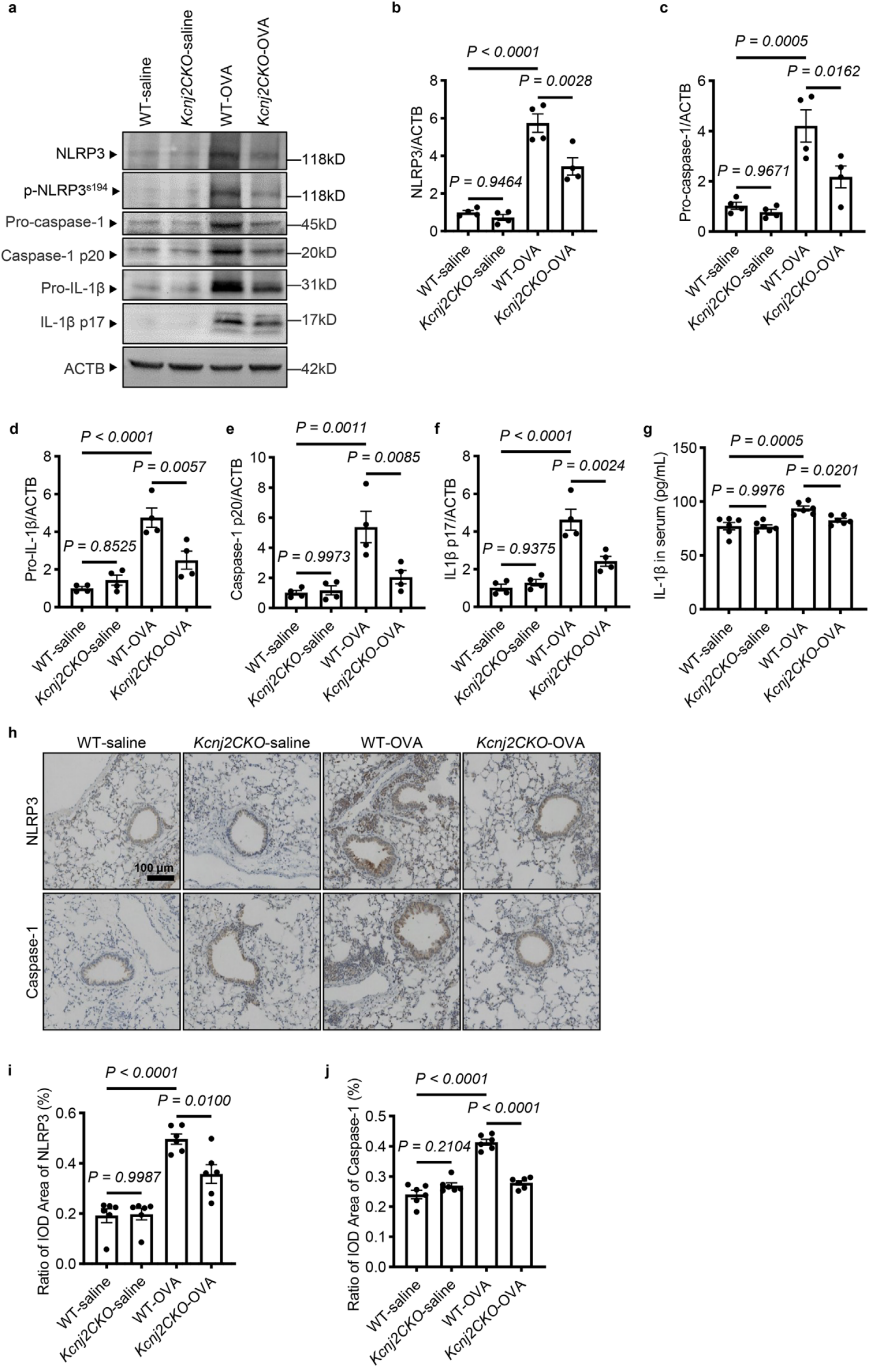
*Kcnj2* deficiency in lung epithelial cells inhibits NLRP3 inflammasome activation in OVA‐induced asthmatic mice. (a) Western blotting for NLRP3, phosphorylated NLRP3 (Phospho‐NLRP3 Ser194), pro‐caspase‐1, pro‐IL‐1β, activated caspase‐1, mature IL‐1β and ACTB in lung tissues of WT‐saline (*n* = 4) and WT‐OVA (*n* = 4) mice, and *Kcnj2CKO*‐saline (*n* = 4) and *Kcnj2CKO*‐OVA (*n* = 4) mice. (b) Quantification of relative NLRP3 levels (as in a). (c) Quantification of relative pro‐caspase‐1 levels (as in a). (d) Quantification of pro‐IL‐1β relative levels (as in a). (e) Quantification of relative activated caspase‐1 levels (as in a). (f) Quantification of relative mature IL‐1β levels (as in a). (g) Protein levels of IL‐1β in serum of WT‐saline (*n* = 6) and WT‐OVA (*n* = 6) mice, and *Kcnj2CKO*‐saline (*n* = 6) and *Kcnj2CKO*‐OVA (*n* = 6) mice by ELISA. (h) Representative images of immunohistochemistry for NLRP3 and caspase‐1 in the lungs of WT‐saline (*n* = 6) and WT‐OVA (*n* = ) mice, and *Kcnj2CKO*‐saline (*n* = 6) and *Kcnj2CKO*‐OVA (*n* = 6) mice. Quantification of average optical density values of NLRP3 (i) and caspase‐1 (j) (as in h). Data are shown mean ± s.d. one‐way ANOVA.

Potassium channels have been reported to function as specific upstream regulators of NLRP3 inflammasome activation, as evidenced in macrophages stimulated by bacterial toxins, particulate matter, or in response to ATP [31, 32, 33, 34, 47]. *NLRP3* global knockout mice exhibit relieved allergic airway inflammation and decreased levels of Th2 cytokines in OVA‐induced allergic asthmatic mice [26]. In hRV16‐infected patients with chronic rhinosinusitis, NLRP3 levels are significantly increased in the epithelium of chronic inflammatory nasal mucosa and are associated with goblet cell hyperplasia [30]. Based on these findings, we hypothesized that deficiency of the inwardly rectifying potassium channel Kcnj2 may impair NLRP3 inflammasome activation in lung epithelial cells in allergic asthmatic conditions. To test this hypothesis, we analyzed changes in NLRP3 inflammasome activation in the lungs of saline‐ and OVA‐sensitized and challenged WT and *Kcnj2CKO* mice. NLRP3 inflammasome activation was evaluated by assessing NLRP3 phosphorylation, caspase‐1 activation (caspase‐1 subunit p20), and IL‐1β maturation and release. OVA sensitization and challenge increased protein levels of NLRP3, pro‐caspase‐1, and pro‐IL‐1β in the lungs compared with WT‐saline mice (Figure 4a–d). Notably, *Kcnj2CKO*‐OVA mice displayed reduced levels of NLRP3, pro‐caspase‐1, and pro‐IL‐1β in the lungs compared with WT‐OVA mice (Figure 4a–d). However, no obvious changes in levels of NLRP3, pro‐caspase‐1, or pro–IL‐1β were observed in the lungs between *Kcnj2CKO*‐saline mice and WT‐saline mice (Figure 4a–d). OVA sensitization and challenge also enhanced NLRP3 inflammasome activation as evidenced by increased NLRP3 phosphorylation at the Ser194 residue (Figure 4a), caspase‐1 activation (Figure 4a,e), and IL‐1β maturation (Figure 4a,f) and release (Figure 4g) in the lungs compared with WT‐saline mice. Notably, *Kcnj2CKO*‐OVA mice displayed reduced NLRP3 inflammasome activation in the lungs compared with WT‐OVA mice (Figure 4a,e–g). Next, we examined protein levels of NLRP3 and caspase‐1 in lung tissues by immunohistochemistry. WT‐OVA mice exhibited increased levels of NLRP3 and caspase‐1 in the lungs, including the airway epithelium and infiltrated inflammatory cells in peribronchial and perivascular regions compared with WT‐saline mice (Figure 4h–j). Consistently, *Kcnj2CKO*‐OVA mice exhibited decreased protein levels of NLRP3 and caspase‐1 in the lungs, including the airway epithelium, compared with WT‐OVA mice (Figure 4h–j). Collectively, these data suggest that KCNJ2 functions in lung epithelial cells to promote NLRP3 inflammasome activation, possibly by enhancing NLRP3 phosphorylation in asthmatic conditions.

### 2.5 KCNJ2 Inhibition by ML133 Attenuates Airway Hyperresponsiveness, Airway Inflammation, Th2 Immune Responses, and Expression of Alarmins in HDM‐Induced Asthmatic Mice

**FIGURE 5:**
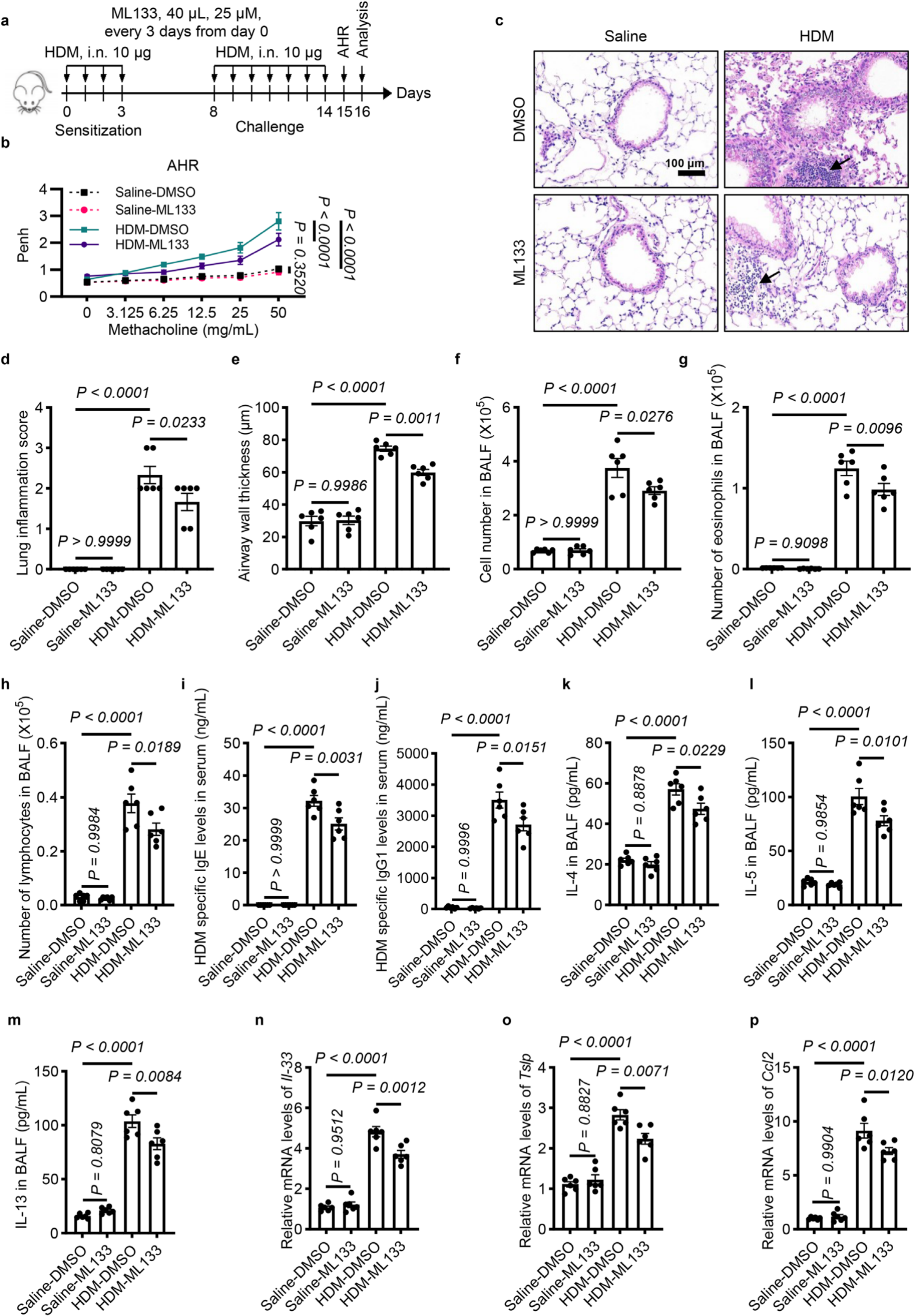
KCNJ2 inhibition by ML133 reduces airway hyperresponsiveness, airway inflammation, and Th2 immune responses in HDM‐induced asthmatic mice. (a) Timeline for the HDM‐induced asthmatic mouse model, indicating time points for sensitization (10 µg HDM), challenge (10 µg HDM), and analysis (i.n.: intranasal). ML133: 40 µL, 25 µM, every 3 days from day 0. (b) Penh measurements in response to increasing doses of aerosolized methacholine in saline‐DMSO (*n* = 6), saline‐ML133 (*n* = 6), HDM‐DMSO (*n* = 6) and HDM‐ML133 (*n* = 6) mice. (c) Representative images of lung tissue sections stained with hematoxylin and eosin from saline‐DMSO (*n* = 6), saline‐ML133 (*n* = 6), HDM‐DMSO (*n* = 6), and HDM‐ML133 (*n* = 6) mice. (d) Quantification of inflammation score (as in c). Arrows point to airway inflammatory cells. (e) Quantification of airway wall thickness (as in c). Numbers of total cells (f), eosinophils (g), and lymphocytes (h) in BALF of saline‐DMSO (*n* = 6), saline‐ML133 (*n* = 6), HDM‐DMSO (*n* = 6), and HDM‐ML133 (*n* = 6) mice. (i) HDM‐specific IgE levels in the serum of saline‐DMSO (*n* = 6), saline‐ML133 (*n* = 6), HDM‐DMSO (*n* = 6) and HDM‐ML133 (*n* = 6) mice. (j) HDM‐specific IgG1 levels in the serum of saline‐DMSO (*n* = 6), saline‐ML133 (*n* = 6), HDM‐DMSO (*n* = 6) and HDM‐ML133 (*n* = 6) mice. Protein levels of IL‐4 (k), IL‐5 (l), and IL‐13 (m) in BALF of saline‐DMSO (*n* = 6), saline‐ML133 (*n* = 6), HDM‐DMSO (*n* = 6), and HDM‐ML133 (*n* = 6) mice as measured by ELISA. RT‐qPCR analysis of *Il‐33* (n), *Tslp* (o) and *Ccl2* (p) in the lungs of saline‐DMSO (*n* = 6), saline‐ML133 (*n* = 6), HDM‐DMSO (*n* = 6), and HDM‐ML133 (*n* = 6) mice. Data are shown as mean ± s.d., two‐way ANOVA (b), one‐way ANOVA (d–p).

We further investigated the effect of KCNJ2 inhibition on AHR, airway inflammation and Th2 immune responses in the HDM (house dust mite)‐induced asthmatic mouse model using ML133, a selective blocker of KCNJ2 (Figure 5a) [48]. Pharmacological inhibition of KCNJ2 by ML133 significantly attenuated AHR in response to increasing doses of methacholine in HDM‐induced asthmatic mice compared with HDM‐DMSO mice (Figure 5b). There were no significant differences in AHR between saline‐DMSO and saline‐ML133 mice (Figure 5b). Next, we evaluated the effect of ML133 on HDM‐induced lung inflammation. HDM‐DMSO mice exhibited marked infiltration of inflammatory cells in the peribronchial and perivascular regions (Figure 5c). Inhibition of KCNJ2 by ML133 significantly reduced inflammatory cell infiltration in HDM‐ML133 mice compared with HDM‐DMSO mice (Figure 5c,d). HDM‐ML133 mice showed decreased airway wall thickness compared with HDM‐DMSO mice (Figure 5e). Consistently, saline‐ML133 mice displayed no obvious changes in the numbers of total cells, eosinophils, or lymphocytes in BALF compared with saline‐DMSO mice (Figure 5f–h). Interestingly, HDM‐ML133 mice exhibited decreased numbers of total cells, eosinophils, and lymphocytes in the BALF compared with HDM‐DMSO mice (Figure 5f–h). HDM sensitization and challenge led to elevated serum IgE and IgG1 levels (Figure 5i,j). HDM‐ML133 mice displayed reduced protein levels of IgE and IgG1 in serum compared with those of HDM‐DMSO mice (Figure 5i,j). We then assessed the impact of Kcnj2 inhibition on Th2 cytokine production. HDM sensitization and challenge caused increased levels of IL‐4, IL‐5, and IL‐13 in the BALF of HDM‐DMSO mice compared with saline‐DMSO mice (Figure 5k–m). Notably, HDM‐ML133 mice displayed decreased levels of IL‐4, IL‐5, and IL‐13 in the BALF compared with those of HDM‐DMSO mice (Figure 5k–m). These data suggest that KCNJ2 also functions to mediate Th2 inflammation in the HDM‐induced asthmatic mouse model.

Next, we examined alarmins, including IL‐33, TSLP, and CCL2. Consistent with our earlier observations, HDM sensitization and challenge led to significantly increased levels of *Il‐33*, *Tslp*, and *Ccl2* in the lungs compared with the saline group (Figure 5n–p). Notably, levels of *Il‐33*, *Tslp*, and *Ccl2* were significantly reduced in the lungs of HDM‐ML133 mice compared with those of HDM‐DMSO mice (Figure 5n–p). However, we found no obvious differences in levels of *Il‐33*, *Tslp*, or *Ccl2* in the lungs between saline‐ML133 mice and saline‐DMSO mice (Figure 5n–p). Collectively, these data indicate that KCNJ2 regulates airway hyperresponsiveness, airway inflammation, Th2 immune responses, and expression of alarmins in asthmatic conditions in an ion channel activity‐dependent manner.

### 2.6 KCNJ2 Inhibition Attenuates Goblet Cell Hyperplasia and Mucus Overproduction in HDM‐Induced Asthmatic Mice

**FIGURE 6:**
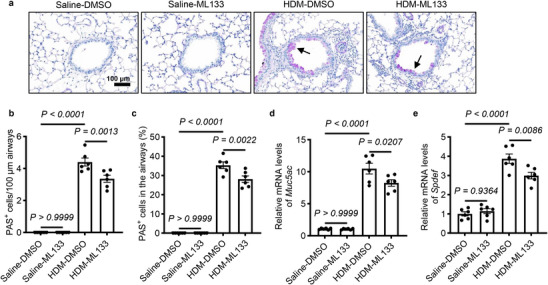
KCNJ2 inhibition by ML133 reduces goblet cell metaplasia and mucus overproduction in HDM‐induced asthmatic mice. (a) Representative images of lung tissue sections stained with PAS from saline‐DMSO (*n* = 6), saline‐ML133 (*n* = 6), HDM‐DMSO (*n* = 6), and HDM‐ML133 (*n* = 6) mice. Arrows point to PAS^+^ goblet cells. (b) Quantification of PAS^+^ cells per 100‐µm airway of saline‐DMSO (*n* = 6), saline‐ML133 (*n* = 6), HDM‐DMSO (*n* = 6), and HDM‐ML133 (*n* = 6) mice. 0, 0, 1585 and 830 PAS ^+^ cells were analyzed for saline‐DMSO (*n* = 6), saline‐ML133 (*n* = 6), HDM‐DMSO (*n* = 6) and HDM‐ML133 (*n* = 6) mice, respectively. (c) Percentage of PAS^+^ cells in the airway epithelium of saline‐DMSO (*n* = 6), saline‐ML133 (*n* = 6), HDM‐DMSO (*n* = 6) and HDM‐ML133 (*n* = 6) mice. RT‐qPCR analysis of *Muc5ac* (d) and *Spdef* (e) in the lungs of saline‐DMSO (*n* = 6), saline‐ML133 (*n* = 6), HDM‐DMSO (*n* = 6) and HDM‐ML133 (*n* = 6) mice. Data are shown as mean ± s.d. one‐way ANOVA.

Next, we examined the effect of ML133 on goblet cell differentiation in the lungs. HDM treatment significantly increased the number of PAS^+^ goblet cells in the airway epithelium (Figure 6a–c). Inhibition of KCNJ2 by ML133 markedly reduced the number of PAS^+^ goblet cells compared with HDM‐DMSO mice (Figure 6a–c). Consistently, ML133 treatment significantly decreased *Muc5ac* mRNA levels in the lungs compared with HDM‐DMSO mice (Figure 6d). However, no significant differences in PAS^+^ cell numbers or *Muc5ac* mRNA levels were observed between saline‐DMSO mice and saline‐ML133 mice (Figure 6a–d). In addition, HDM sensitization and challenge led to increased mRNA levels of *Spdef* in the lungs of HDM‐DMSO mice compared with saline‐DMSO mice (Figure 6e). Notably, *Spdef* levels in the lungs were significantly reduced in HDM‐ML133 mice compared with HDM‐DMSO mice, but not between saline‐DMSO mice and saline‐ML133 mice (Figure 6e). Altogether, these data suggest that KCNJ2 promotes goblet cell hyperplasia and mucus overproduction in asthmatic conditions in an ion channel activity‐dependent manner.

### 2.7 KCNJ2 Overexpression Exacerbates Airway Hyperresponsiveness, Airway Inflammation, Th2 Immune Responses, and Expression of Alarmins in HDM‐Induced Asthmatic Mice

**FIGURE 7:**
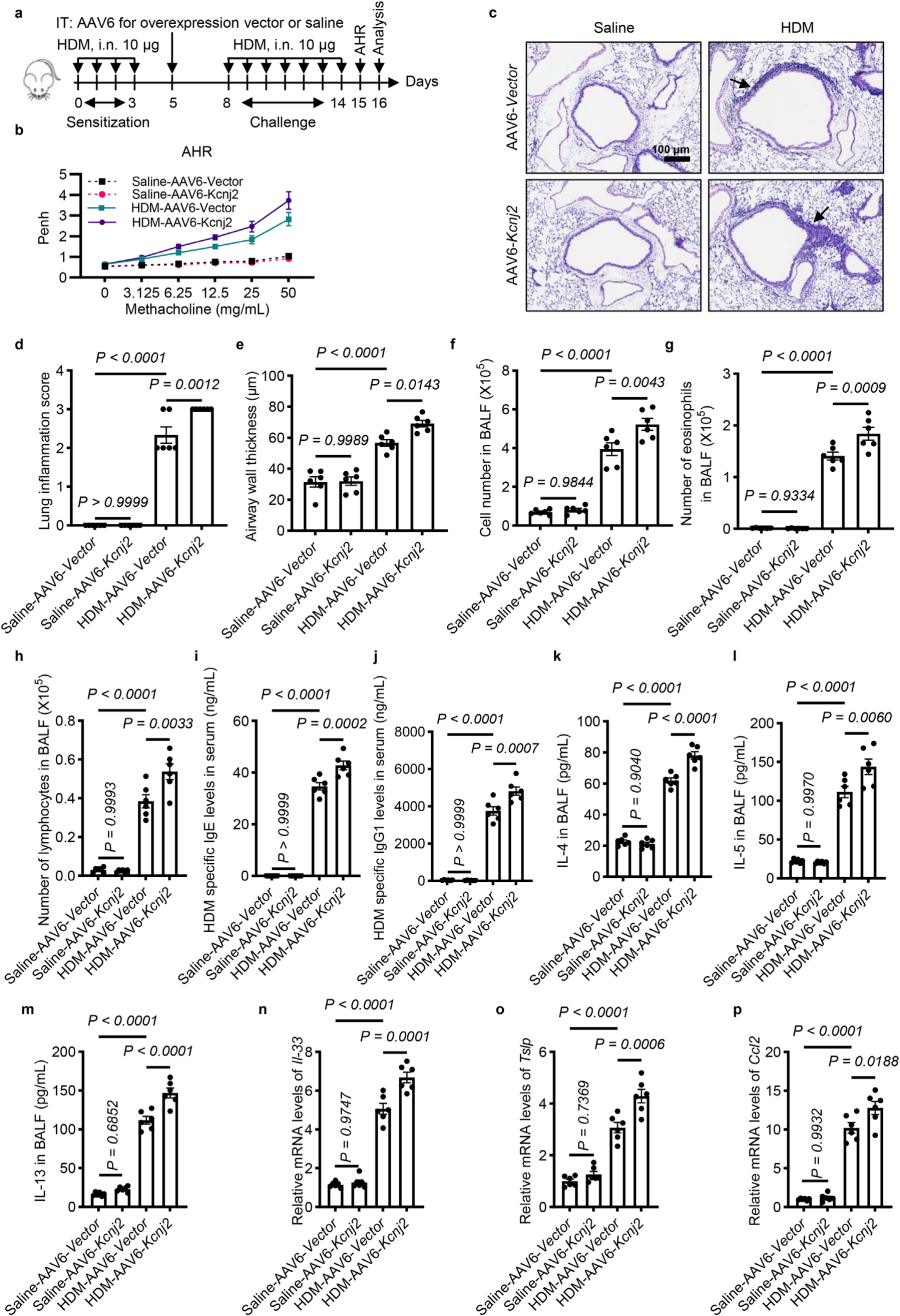
KCNJ2 overexpression promotes airway hyperresponsiveness, airway inflammation, Th2 immune responses, and expression of alarmins in HDM‐induced asthmatic mice. (a) Timeline for the HDM‐induced asthmatic mouse model, indicating time points for sensitization (10 µg HDM), challenge (10 µg HDM), and analysis (i.n.: intranasal). ML133: 40 µL, 25 µM, every 3 days from day 0, AAV6 virus airway injection on day 5. (b) Penh measurements in response to increasing doses of aerosolized methacholine in saline‐AAV6‐Vector (*n* = 6), saline‐AAV6‐*Kcnj2* (*n* = 6), HDM‐AAV6‐Vector (*n* = 6), and HDM‐AAV6‐*Kcnj2* (*n* = 6) mice. (c) Representative images of lung tissue sections stained with hematoxylin and eosin from saline‐AAV6‐Vector (*n* = 6), saline‐AAV6‐*Kcnj2* (*n* = 6), HDM‐AAV6‐Vector (*n* = 6), and HDM‐AAV6‐*Kcnj2* (*n* = 6) mice. Arrows point to airway inflammatory cells. (d) Quantification of inflammation score (as in c). (e) Quantification of airway wall thickness (as in c). Numbers of total cells (f), eosinophils (g), and lymphocytes (h) in BALF of saline‐AAV6‐Vector (*n* = 6), saline‐AAV6‐*Kcnj2* (*n* =  6), HDM‐AAV6‐Vector (*n* = 6), and HDM‐AAV6‐*Kcnj2* (*n* = 6) mice. (i) HDM‐specific IgE levels in the serum of saline‐AAV6‐Vector (*n* = 6), saline‐AAV6‐*Kcnj2* (*n* = 6), HDM‐AAV6‐Vector (n = 6), and HDM‐AAV6‐*Kcnj2* (*n* = 6) mice. (j) HDM‐specific IgG1 levels in the serum of saline‐AAV6‐Vector (*n* = 6), saline‐AAV6‐*Kcnj2* (*n* = 6), HDM‐AAV6‐Vector (*n* = 6), and HDM‐AAV6‐*Kcnj2* (*n* = 6) mice. Protein levels of IL‐4 (k), IL‐5 (l) and IL‐13 (m) in BALF of saline‐AAV6‐Vector (*n* = 6), saline‐AAV6‐*Kcnj2* (*n* = 6), HDM‐AAV6‐Vector (*n* = 6), and HDM‐AAV6‐*Kcnj2* (*n* = 6) mice as measured by ELISA. RT‐qPCR analysis of *Il‐33* (n), *Tslp* (o) and *Ccl2* (p) in the lungs of saline‐AAV6‐Vector (*n* = 6), saline‐AAV6‐*Kcnj2* (*n* = 6), HDM‐AAV6‐Vector (*n* = 6), and HDM‐AAV6‐*Kcnj2* (*n* = 6) mice. Data are shown as mean ± s.d., two‐way ANOVA (b), one‐way ANOVA (d–p).

We further explored the effect of KCNJ2 overexpression on AHR, airway inflammation, and Th2 immune responses in HDM‐induced asthmatic mice. Because adeno‐associated virus type 6 (AAV6) exhibits a high transduction efficiency in lung epithelial cells, especially in the airway epithelium in mice [49, 50], we overexpressed *Kcnj2* in the lungs via intratracheal instillation of the AAV6‐packed vector to stably express *Kcnj2* (AAV6 [ssAAV. CAG. mKcnj2‐P2A‐EGFP. WPRE. SV40pA]) (Figure 7a). As expected, AAV6‐mediated transduction led to significant upregulation of *Kcnj2* (Figure S4a,b). KCNJ2 overexpression (AAV6‐*Kcnj2*) significantly enhanced AHR in response to methacholine in HDM‐induced asthmatic mice compared with HDM‐AAV6‐Vector mice (Figure 7b). No significant differences in AHR were observed between saline‐AAV6‐Vector and saline‐AAV6‐*Kcnj2* mice (Figure 7b). Next, we evaluated the effect of KCNJ2 overexpression on HDM‐induced lung inflammation. HDM‐AAV6‐Vector mice exhibited marked infiltration of inflammatory cells in peribronchial and perivascular regions (Figure 7c). Notably, HDM‐AAV6‐*Kcnj2* mice exhibited significantly increased inflammatory cell infiltration in the lungs compared with HDM‐AAV6‐Vector mice (Figure 7c,d). HDM‐AAV6‐*Kcnj2* mice also displayed increased airway wall thickness compared with HDM‐AAV6‐Vector mice (Figure 7e). Consistently, saline‐AAV6‐*Kcnj2* mice showed no significant changes in the numbers of total cells, eosinophils, or lymphocytes in the BALF compared with saline‐AAV6‐Vector mice (Figure 7f–h). Notably, HDM‐AAV6‐*Kcnj2* mice exhibited increased numbers of total cells, eosinophils, and lymphocytes in the BALF compared with HDM‐AAV6‐Vector mice (Figure 7f–h). In addition, HDM‐AAV6‐*Kcnj2* mice displayed increased levels of IgE and IgG1 in serum compared with HDM‐AAV6‐Vector mice (Figure 7i,j). We further assessed the effect of KCNJ2 overexpression on Th2 cytokine production. HDM sensitization and challenge increased levels of IL‐4, IL‐5, and IL‐13 in the BALF of HDM‐AAV6‐Vector mice compared with saline‐AAV6‐Vector mice (Figure 7k–m). Importantly, KCNJ2 overexpression further augmented IL‐4, IL‐5, and IL‐13 production in the BALF of HDM‐challenged mice (Figure 7k–m).

We next examined alarmins, including IL‐33, TSLP, and CCL2. HDM sensitization and challenge significantly increased levels of *Il‐33*, *Tslp* and *Ccl2* in the lungs of HDM‐AAV6‐Vector mice compared with saline‐AAV6‐Vector mice (Figure 7n–p). Notably, levels of *Il‐33*, *Tslp*, and *Ccl2* were significantly elevated in the lungs of HDM‐AAV6‐*Kcnj2* mice compared with HDM‐AAV6‐Vector mice (Figure 7n–p). However, no significant differences in levels of *Il‐33*, *Tslp*, or *Ccl2* were observed in the lungs between saline‐AAV6‐*Kcnj2* and saline‐AAV6‐Vector mice (Figure 7n–p). Collectively, these data indicate that KCNJ2 overexpression exacerbates airway hyperresponsiveness, airway inflammation, Th2 immune responses, and expression of alarmins in HDM‐induced asthmatic conditions.

### 2.8 KCNJ2 Overexpression Enhances Goblet Cell Hyperplasia and Mucus Overproduction in HDM‐Induced Asthmatic Mice

**FIGURE 8:**
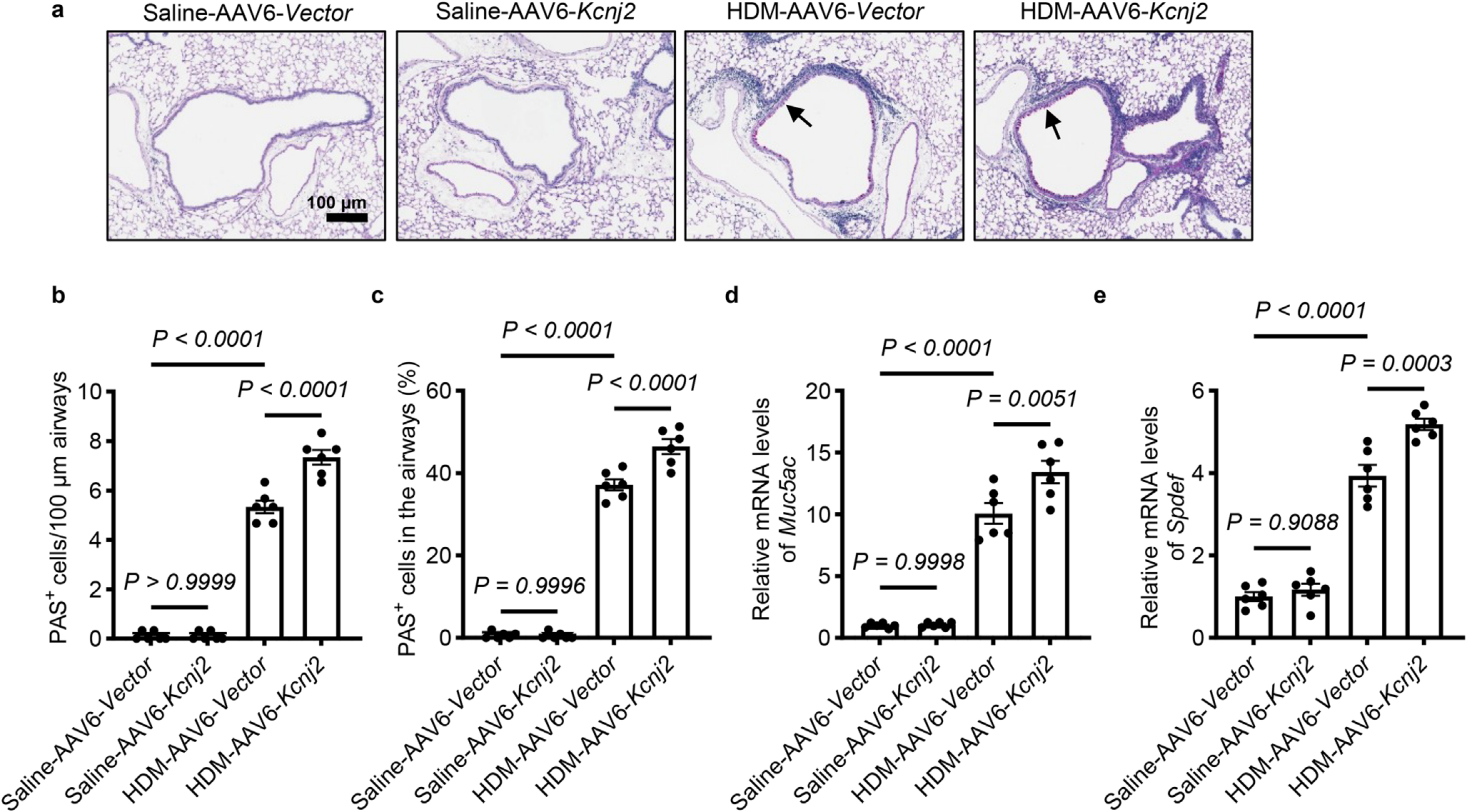
KCNJ2 overexpression promotes goblet cell hyperplasia and mucus overproduction in HDM‐induced asthmatic mice. (a) Representative images of lung tissue sections stained with PAS from saline‐AAV6‐Vector (*n* = 6), saline‐AAV6‐*Kcnj2* (*n* = 6), HDM‐AAV6‐Vector (*n* = 6), and HDM‐AAV6‐*Kcnj2* (*n* = 6) mice. Arrows point to PAS^+^ goblet cells. (b) Quantification of PAS^+^ cells per 100‐µm airway of saline‐AAV6‐Vector (*n* = 6), saline‐AAV6‐*Kcnj2* (*n* = 6), HDM‐AAV6‐Vector (*n* = 6), and HDM‐AAV6‐*Kcnj2* (*n* = 6) mice. 0, 0, 1338 and 1672 PAS ^+^ cells were analyzed for saline‐AAV6‐Vector (*n* = 6), saline‐AAV6‐*Kcnj2* (*n* = 6), HDM‐AAV6‐Vector (*n* = 6), and HDM‐AAV6‐*Kcnj2* (*n* = 6) mice, respectively. (c) Percentage of PAS^+^ cells in the airway epithelium of saline‐AAV6‐Vector (*n* = 6), saline‐AAV6‐*Kcnj2* (*n* = 6), HDM‐AAV6‐Vector (*n* = 6), and HDM‐AAV6‐*Kcnj2* (*n* = 6) mice. RT‐qPCR analysis of *Muc5ac* (d) and *Spdef* (e) in the lungs of saline‐AAV6‐Vector (*n* = 6), saline‐AAV6‐*Kcnj2* (*n* = 6), HDM‐AAV6‐Vector (*n* = 6), and HDM‐AAV6‐*Kcnj2* (*n* = 6) mice. Data are shown as mean ± s.d., one‐way ANOVA.

We further evaluated the impact of KCNJ2 overexpression on goblet cell differentiation and mucus production in the lungs. HDM treatment significantly increased the number of PAS^+^ goblet cells in the airway epithelium (Figure 8a–c). KCNJ2 overexpression further increased PAS^+^ goblet cell numbers compared with HDM‐AAV6‐Vector mice (Figure 8a–c). Consistently, KCNJ2 overexpression also significantly increased *Muc5ac* mRNA levels in the lungs compared with HDM‐AAV6‐Vector mice (Figure 8d). However, no significant differences in PAS^+^ cell numbers or *Muc5ac* mRNA levels were observed between saline‐AAV6‐Vector mice and saline‐KCNJ2 overexpression mice (Figure 8a–d). Additionally, HDM sensitization and challenge led to elevated mRNA levels of *Spdef* in the lungs of HDM‐AAV6‐Vector mice compared with saline‐AAV6‐Vector mice (Figure 8e). Notably, *Spdef* levels in the lungs were further increased in HDM‐AAV6‐*Kcnj2* mice compared with HDM‐AAV6‐Vector mice, but not between saline‐AAV6‐Vector mice and saline‐AAV6‐*Kcnj2* mice (Figure 8e). Collectively, these data suggest that KCNJ2 overexpression promotes goblet cell hyperplasia and mucus overproduction in asthmatic conditions.

### 2.9 KCNJ2 is Required for NLRP3 Inflammasome Activation by Regulating both Ca^2+^ Influx and K^+^ Efflux

**FIGURE 9:**
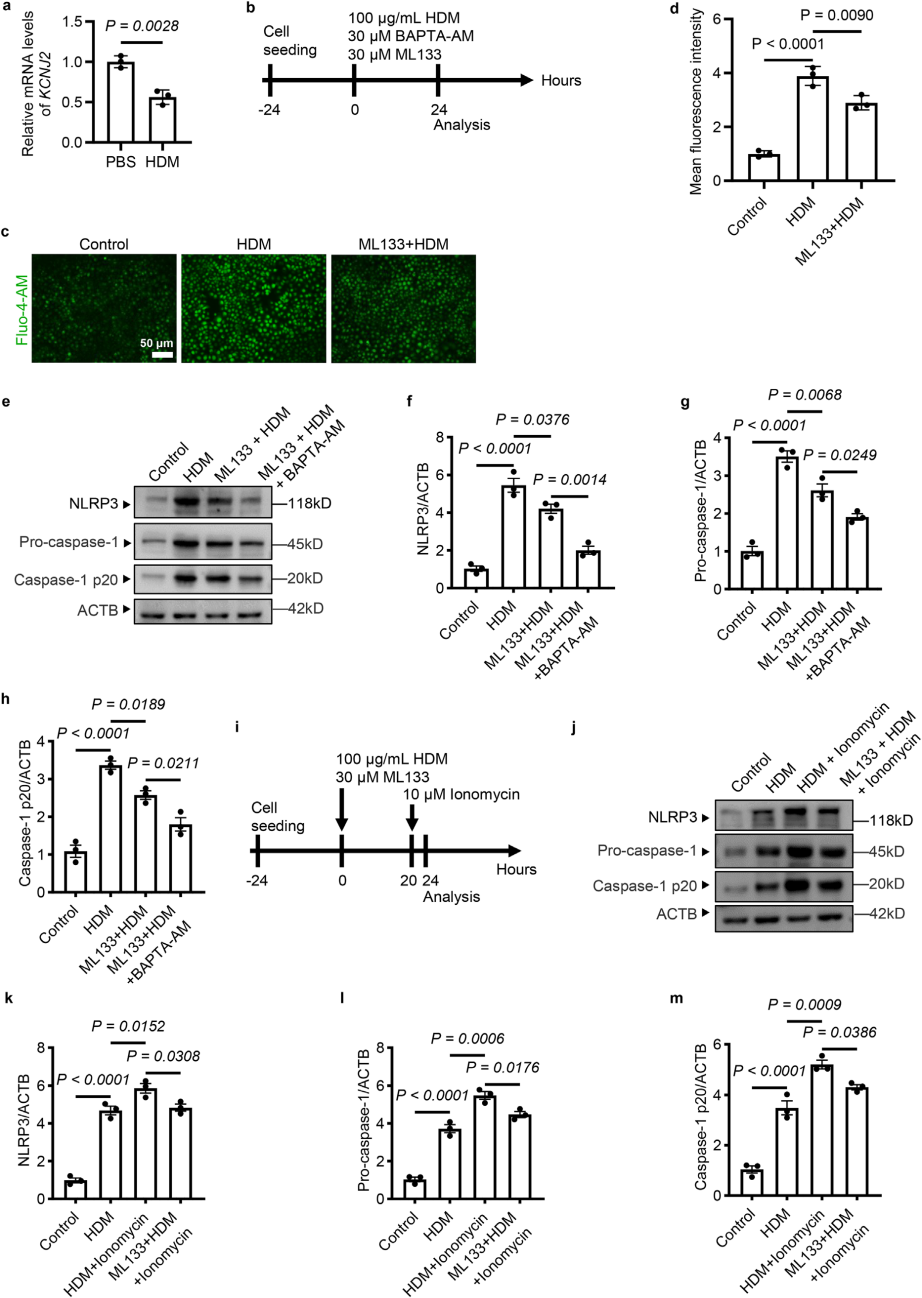
KCNJ2 inhibition decreases intracellular Ca^2+^ levels and NLRP3 inflammasome activation elevated by HDM exposure in airway epithelial cells. (a) RT‐qPCR analysis of *KCNJ2* in PBS and HDM‐treated 16HBE cells. b Timeline for ML133, HDM, and BAPTA‐AM administration. c Fluo‐4 AM imaging in 16HBE cells after PBS, 100 µg/mL HDM, and 30 µM ML133 plus 100 µg/mL HDM treatment. (d) Quantification of mean Fluo‐4 AM fluorescence intensity in 16HBE cells (as in c). (e) Western blotting for NLRP3, pro‐caspase‐1, activated caspase‐1, and ACTB in 16HBE cells after PBS, 100 µg/mL HDM, 30 µM ML133 plus 100 µg/mL HDM, and 30 µM ML133 plus 100 µg/mL HDM plus 30 µM BAPTA‐AM treatment.(f) Quantification of relative NLRP3 levels (as in e). (g) Quantification of relative pro‐caspase‐1 levels (as in e). (h) Quantification of relative activated caspase‐1 levels (as in e). (i) Timeline for ML133, HDM, and Ionomycin administration. (j) Western blotting for NLRP3, pro‐caspase‐1, activated caspase‐1, and ACTB in 16HBE cells after PBS, 100 µg/mL HDM, 100 µg/mL HDM plus 10 µM Ionomycin, and 30 µM ML133 plus 100 µg/mL HDM plus 10 µM Ionomycin treatment. (k) Quantification of relative NLRP3 levels (as in j). (l) Quantification of relative pro‐caspase‐1 levels (as in j). (m) Quantification of relative activated caspase‐1 levels (as in j). Data are shown as mean ± s.d. Unpaired Student's *t*‐test (a), one‐way ANOVA (d, f–h, k–m).

We sought to investigate how KCNJ2 mediates NLRP3 inflammasome activation in asthmatic conditions. We used house dust mite (HDM)‐stimulated 16HBE cells, a human bronchial epithelial cell line, as a model of airway pathological changes and response in asthmatic conditions [51, 52]. Intracellular Ca^2+^ levels have been shown to be critical for NLRP3 inflammasome activation [35]. We hypothesized that KCNJ2 might mediate intracellular Ca^2+^ levels to regulate NLRP3 inflammasome activation in asthmatic conditions. HDM exposure led to reduced *KCNJ2* levels in 16HBE cells (Figure 9a). *KCNJ2* inhibition by ML133 significantly attenuated the HDM exposure‐induced increase in intracellular Ca^2+^ levels in 16HBE cells (Figure 9b‐d). ML133 treatment led to decreased protein levels of NLRP3 (Figure 9e,f) and pro‐caspase‐1 (Figure 9e,g), and reduced NLRP3 inflammasome activation (Figure 9e,h) in HDM‐exposed 16HBE cells. Notably, treatment with BAPTA‐AM, a cell‐permeable Ca^2+^ chelator, further decreased protein levels of NLRP3 and pro‐caspase‐1, and suppressed NLRP3 inflammasome activation caused by HDM exposure (Figure 9e–h). Consistently, induction of intracellular Ca^2+^ by ionomycin further increased protein levels of NLRP3 (Figure 9j,k) and pro‐caspase‐1 (Figure 9j,l), and NLRP3 inflammasome activation was elevated by HDM exposure in 16HBE cells (Figure 9j, m). Notably, ML133 treatment significantly attenuated these effects in HDM+ionomycin‐treated 16HBE cells (Figure 9j–m). These results suggest that KCNJ2‐mediated Ca^2+^ influx is essential for NLRP3 inflammasome activation in airway epithelial cells in asthmatic conditions.

**FIGURE 10:**
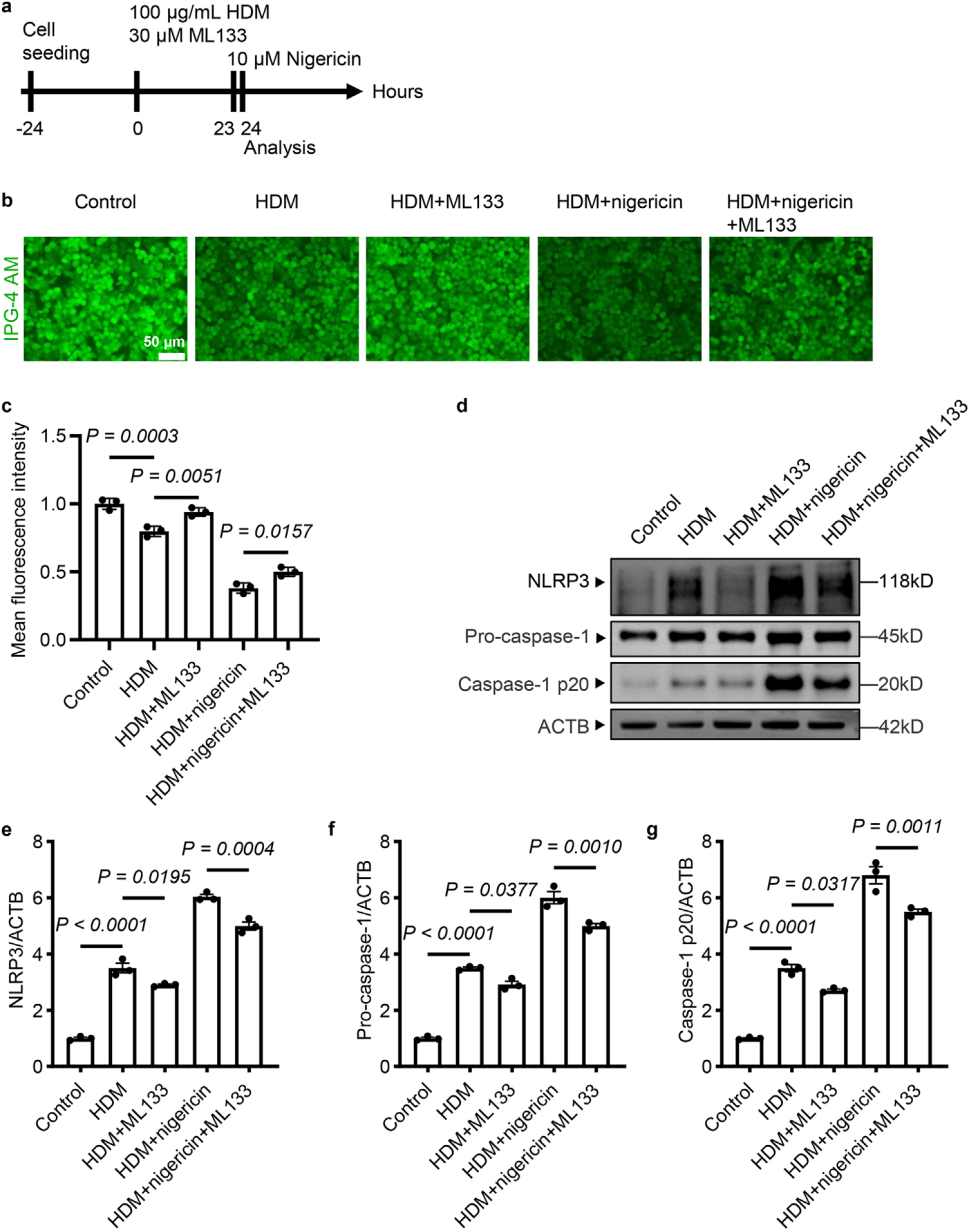
KCNJ2 inhibition increases intracellular K^+^ levels and NLRP3 inflammasome activation, elevated by HDM exposure in airway epithelial cells. (a) Timeline for ML133, HDM, and Nigericin administration. (b) IPG‐4 AM imaging in 16HBE cells after PBS, 100 µg/mL HDM, 100 µg/mL HDM plus 30 µM ML133, 30 µM ML133 plus 10 µM Nigericin, and 100 µg/mL HDM plus 30 µM ML133 plus 10 µM Nigericin treatment. (c) Quantification of mean IPG‐4 AM fluorescence intensity in 16HBE cells (as in b). (d) Western blotting for NLRP3, pro‐caspase‐1, activated caspase‐1, and ACTB in 16HBE cells after PBS, 100 µg/mL HDM, 100 µg/mL HDM plus 30 µM ML133, 100 µg/mL HDM plus 10 µM Nigericin, and 100 µg/mL HDM plus 30 µM ML133 plus 10 µM Nigericin treatment. (e) Quantification of relative NLRP3 levels (as in d). f Quantification of relative pro‐caspase‐1 levels (as in d). (g) Quantification of relative activated caspase‐1 levels (as in d). Data are shown as mean ± s.d., one‐way ANOVA.

Intracellular K^+^ levels are also important for NLRP3 inflammasome activation. We hypothesized that KCNJ2 may also regulate NLRP3 inflammasome activation through modulation of K^+^ efflux in asthmatic conditions. HDM exposure decreased intracellular K^+^ levels in 16HBE cells compared with controls (Figure 10a–c). Inhibition of KCNJ2 by 30 µM ML133 partially restored intracellular K^+^ levels in HDM‐treated cells (Figure 10a–c). Nigericin, a bacterial pore‐forming toxin known to induce a marked depletion of intracellular K^+^ [32, 53]. Notably, treatment with 10 µM nigericin further decreased intracellular K^+^ levels in HDM‐exposed 16HBE cells (Figure 10a–c). As expected, nigericin treatment further increased HDM exposure‐induced elevation of protein levels of NLRP3 (Figure 10d,e), pro‐caspase‐1 (Figure 10d,f), and caspase‐1 p20 (Figure 10d,g). Importantly, ML133 treatment partially attenuated this effect in the HDM+nigericin+ML133 group (Figure 10d–g). These results indicate that KCNJ2‐mediated K^+^ efflux is also involved in NLRP3 inflammasome activation in airway epithelial cells in asthmatic conditions.

### 2.10 KCNJ2 Inhibition Leads to Reduced Goblet Cell Differentiation and Mucus Production in a NLRP3 Inflammasome‐Dependent Manner in Airway Epithelial Cells of Asthmatic Patients

**FIGURE 11:**
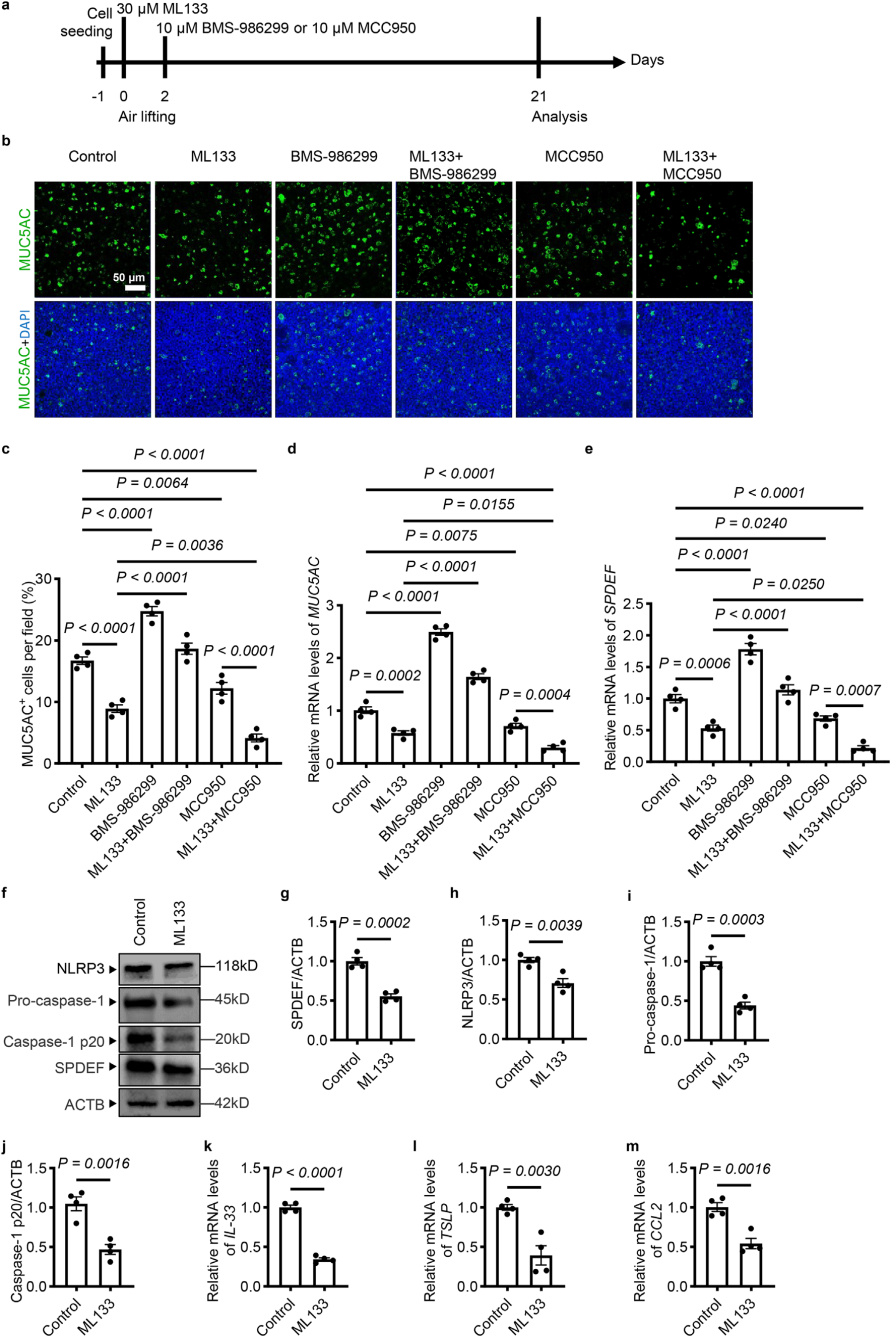
NLRP3 inflammasome activity functions downstream of KCNJ2 in regulating goblet cell differentiation and mucus production in airway epithelial cells of asthmatic patients. (a) Timeline for ML133, BMS‐986299, and MCC950 administration. (b) Immunostaining for MUC5AC (green) and DAPI staining (blue) in pHBE cells from asthmatic patients at the ALI after 21 days of DMSO (*n* = 4) or 30 µM ML133 (*n* = 4) treatment, 19 days of 10 µM BMS‐986299 treatment (*n* = 4), 21 days of 30 µM ML133 plus 19 days of 10 µM BMS‐986299 treatment (*n* = 4), 19 days of 10 µM MCC950 treatment (*n* = 4), 21 days of 30 µM ML133 plus 19 days of 10 µM MCC950 treatment (*n* = 4). (c) Percentage of MUC5AC^+^ goblet cells in pHBE cells at the ALI (as in b). 6684, 3567, 9901, 7462, 4890 and 1654 MUC5AC^+^ goblet cells were analyzed for DMSO (*n* = 4) or 30 µM ML133 (*n* = 4) treatment, 19 days of 10 µM BMS‐986299 treatment (*n* = 4), 21 days of 30 µM ML133 plus 19 days of 10 µM BMS‐986299 treatment (*n* = 4), 19 days of 10 µM MCC950 treatment (*n* = 4), 21 days of 30 µM ML133 plus 19 days of 10 µM MCC950 treatment (*n* = 4), respectively. RT‐qPCR analysis of *MUC5AC* (d) and *SPDEF* (e) in pHBE cells at the ALI (as in b). (f) Western blotting for NLRP3, pro‐caspase‐1, activated caspase‐1, SPDEF, and ACTB in pHBE cells from asthmatic patients at the ALI after 21 days of DMSO (*n* = 4) or 30 µM ML133 (*n* = 4) treatment. (g) Quantification of relative SPDEF levels (as in f). (h) Quantification of relative NLRP3 levels (as in f). (i) Quantification of relative pro‐caspase‐1 levels (as in f). (j) Quantification of relative activated caspase‐1 levels (as in f). RT‐qPCR analysis of *IL‐33* (k), *TSLP* (l) and *CCL‐2* (m) in pHBE cells from asthmatic patients at the ALI after 21 days of DMSO (*n* = 4) or 30 µM ML133 (*n* = 4) treatment. Data are shown as mean ± s.d. one‐way ANOVA (c–e), Unpaired Students’ *t*‐test (g–m).

To examine whether KCNJ2 regulates airway goblet cell differentiation and mucus production in asthmatic patients, we utilized primary human bronchial epithelial (pHBE) cells from asthmatic patients cultured in vitro at an air–liquid interface (ALI) as a model of the airway epithelium, which can mimic its in vivo structure and pathology in asthmatics (Table S2) [54, 55]. Undifferentiated pHBE cells were seeded onto transwell filters, and differentiation was initiated by removing the apical medium (defined as day 0). Samples were harvested on day 21 for analysis (Figure 11a and Figure S5). ALI cultures exhibited a decreased number of MUC5AC^+^ goblet cells (Figure 11b,c) and reduced levels of *MUC5AC* (Figure 11d) and *SPDEF* (Figure 11e) after treatment with ML133. However, ML133 treatment appeared to cause no significant differences in pHBE cell proliferation (Figure S6), indicating that the reduction in goblet cells was not due to impaired cell growth. ML133 treatment decreased protein levels of SPDEF, NLRP3, and pro‐caspase‐1 in pHBE cells from asthmatic patients in ALI cultures (Figure 11f–i). Notably, ML133 treatment also attenuated NLRP3 inflammasome activation (Figure 11f,j). Interestingly, levels of alarmins, including *IL‐33*, *TSLP*, and *CCL2* were also decreased in pHBE cells after ML133 treatment (Figure 11k–m). Next, we examined the effect of NLRP3 inflammasome inhibition and activation on goblet cell differentiation and mucus production. ALI cultures treated by MCC950, a selective NLRP3 inhibitor [56], displayed a reduced number of MUC5AC^+^ goblet cells (Figure 11b,c) and decreased levels of *MUC5AC* (Figure 11d). Interestingly, MCC950 treatment also decreased *SPDEF* levels compared with controls (Figure 11e). Correspondingly, pharmacological activation of NLRP3 by BMS‐986299, a NLRP3 agonist [57], led to increased numbers of MUC5AC^+^ goblet cells (Figure 11b,c) and elevated levels of *MUC5AC* (Figure 11d) and *SPDEF* (Figure 11e). Notably, BMS‐986299 treatment partially reversed the inhibition effects of ML133 on goblet cell differentiation (Figure 11b,c) and expression of *MUC5AC* (Figure 11d) and *SPDEF* (Figure 11e) in ALI cultures, indicating that NLRP3 acts downstream of KCNJ2 in mediating airway goblet cell differentiation and mucus production in the asthmatic airway epithelium. Moreover, combined ML133 and MCC950 treatment further suppressed MUC5AC^+^ goblet cell differentiation (Figure 11b,c) and expression of *MUC5AC* (Figure 11d) and *SPDEF* (Figure 11e) compared with MCC950 treatment. To validate these findings in a three‐dimensional model, we generated airway organoids derived from pHBE cells of asthmatic patients, a model that can mimic the structure and pathological changes of the airway epithelium in asthmatics [58]. Consistent with the ALI results, a treatment with ML133 significantly reduced the number of MUC5AC^+^ goblet cells in these airway organoids compared with controls (Figure S7a–c). MCC950 treatment also caused a decreased number of MUC5AC^+^ goblet cells in these airway organoids (Figure S7a–c). Altogether, these results suggest that KCNJ2 inhibition suppresses goblet cell differentiation and mucus production in airway epithelial cells from patients with asthma, at least in part by attenuating NLRP3 inflammasome activation and downstream SPDEF expression.

### 2.11 KCNJ2 Inhibition Dampens Goblet Cell Differentiation and Mucus Production in a NLRP3 Inflammasome‐Dependent Manner in Response to IL‐13 in Human Airway Epithelial Cells

**FIGURE 12:**
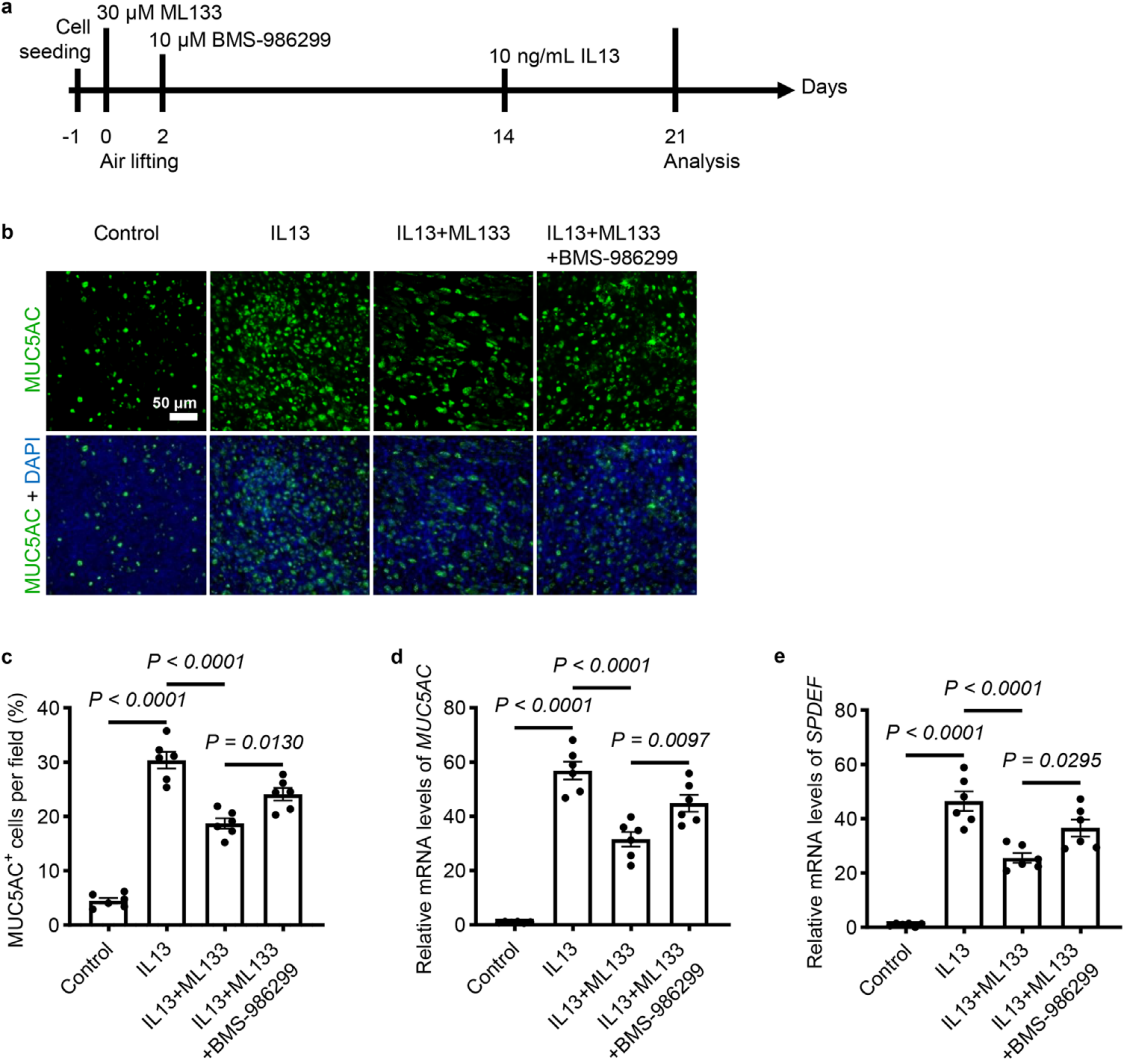
NLRP3 inflammasome activation dampens KCNJ2 inhibition‐regulated IL‐13‐induced goblet cell hyperplasia and mucus production. (a) Timeline for ML133, BMS‐986299, and IL‐13 administration. (b) Immunostaining for MUC5AC (green) and DAPI staining (blue) in pHBE cells from healthy donors at the ALI after 21 days of DMSO (*n* = 4) treatment, 7 days of 10 ng/mL IL‐13 treatment (*n* = 4), 21 days of 30 µM ML133 plus 7 days of 10 ng/mL IL‐13 treatment (*n* = 4), 21 days of 30 µM ML133 plus 19 days of 10 µM BMS‐986299 and 7 days of 10 ng/mL IL‐13 treatment (*n* = 4). (c) Percentage of MUC5AC^+^ goblet cells in pHBE cells at the ALI (as in b). 1842, 12481, 7694 and 9901 MUC5AC^+^ goblet cells were analyzed for DMSO (*n* = 4) treatment, 7 days of 10 ng/mL IL‐13 treatment (*n* = 4), 21 days of 30 µM ML133 plus 7 days of 10 ng/mL IL‐13 treatment (*n* = 4), 21 days of 30 µM ML133 plus 19 days of 10 µM BMS‐986299 and 7 days of 10 ng/mL IL‐13 treatment (*n* = 4), respectively. RT‐qPCR analysis of *MUC5AC* (d) and *SPDEF* (e) in pHBE cells at the ALI. Data are shown as mean ± s.d. one‐way ANOVA.

IL‐13 is a key Th2 cytokine that drives airway remodeling and mucus production in asthma. Next, we investigated whether KCNJ2 inhibition modulates on goblet cell differentiation and mucus production in response to IL‐13 in human airway epithelial cells from healthy donors cultured at the ALI (Figure 12a). IL‐13 treatment led to goblet cell hyperplasia (Figure 12b,c), mucus overproduction (Figure 12b), and increased mRNA levels of *MUC5AC* (Figure 12d) and *SPDEF* (Figure 12e) in in vitro ALI‐cultured pHBE cells from healthy donors, similar to previously reported studies [59, 60, 61]. After treatment with ML133, IL‐13‐treated ALI cultures exhibited a decreased number of MUC5AC^+^ goblet cells (Figure 12b,c) and reduced mRNA levels of *MUC5AC* (Figure 12d) and *SPDEF* (Figure 12e) compared with controls. Interestingly, activation of NLRP3 by BMS‐986299 partially attenuated the inhibition of goblet cell differentiation and expression of *MUC5AC* and *SPDEF* caused by ML133 treatment in IL‐13‐treated ALI cultures. Collectively, these data suggest that KCNJ2 regulates IL‐13‐induced goblet cell differentiation and mucus production in airway epithelial cells, at least in part through a NLRP3 inflammasome dependent‐manner.

## 3 Discussion

Pharmacological studies have identified two potassium channels as potential therapeutic targets for asthma [62, 63, 64]. In two GWAS studies, SNPs in *KCNJ2* are associated with asthma susceptibility [20, 65]. *KCNJ2* has also been reported to be associated with bronchodilator responsiveness in chronic obstructive pulmonary disease and with pulmonary function [18, 19, 66]. Here, we show that asthmatic patients exhibit decreased KCNJ2 levels, which are associated with goblet cell hyperplasia and MUC5AC overproduction in the airways. KCNJ2 inhibition reduces the number of MUC5AC^+^ goblet cells and decreases MUC5AC levels in both OVA‐ and HDM‐induced asthmatic mouse airways, as well as in pHBE cells derived from asthmatic patients. In addition, KCNJ2 inhibition alleviates OVA‐induced airway inflammation and suppresses Th2 cytokines production in OVA‐induced asthmatic mice. Moreover, KCNJ2 inactivation inhibits alarmin production in asthmatic mice and pHBE cells of asthmatic patients. Collectively, these findings indicate that KCNJ2 plays a deleterious role in airway inflammation and remodeling. Given that more than 30 potassium channels have been reported to be expressed in the respiratory epithelium [67], it will be of considerable interest to test whether other member(s) play a role in asthmatic airway inflammation and/or remodeling.

Changes in KCNJ2 expression exhibit a negative correlation with goblet cell hyperplasia and MUC5AC production in asthmatic conditions in our study. It is possible that animals and/or humans use compensatory or buffering mechanisms to mitigate the severity of pulmonary disease in some context. In support of this concept, expression of *KCNJ15*, another member of the inwardly rectifying potassium channel family, has been reported to negatively correlate with its function in *Mycobacterium tuberculosis* (*Mtb*) growth control upon *Mtb* infection, suggesting a protective host response [68]. Accordingly, KCNJ2 downregulation in asthmatic conditions may represent an adaptive or protective response to prevent the development of more severe symptoms of asthma. Our work shows that KCNJ2 expression is downregulated in the lungs of asthmatic patients. *KCNJ2* mRNA levels appear slightly downregulated in asthmatic patients in GSE43696, GSE63142, and GSE179156. KCNJ2 protein levels in the airways in asthmatic patients decrease to around 70% of those in healthy donors in immunochemistry. MUC5AC levels are increased in these asthmatic patients compared with healthy donors. In functional analysis, we find that epithelial deletion of KCNJ2 or its inhibition by ML133 suppresses MUC5AC expression. Because KCNJ2 deletion or its inhibition by ML133 can markedly block its activity, it is possible that a slight or mild downregulation of KCNJ2 is insufficient to counteract MUC5AC overproduction in asthmatic patients. Therefore, the partial downregulation of KCNJ2 may represent a failing attempt to prevent MUC5AC overproduction in asthmatic patients. Because *Kcnj2* expression can be regulated by several factors [69, 70], it remains possible that *Kcnj2* expression is regulated by some signaling pathway(s) or gene(s) in asthma development. Identification of the upstream regulators of *Kcnj2* may therefore provide further mechanistic insight into its role in airway inflammation and remodeling.

NLRP3 inflammasome activation has been shown to be involved in several respiratory diseases, including asthma, chronic obstructive pulmonary disease, and chronic rhinosinusitis. For example, NLRP3 levels are markedly increased in the epithelium of chronic inflammatory nasal mucosa from hRV16‐infected patients with chronic rhinosinusitis and are associated with goblet cell hyperplasia [30]. Although global deficiency of NLRP3 inhibits goblet cell hyperplasia in the airways in the asthmatic mouse model [26], it remains unclear whether this effect is due to a direct inhibition of goblet cell differentiation or is secondary to reduced lung inflammation. In the present study, we show that pharmacological inhibition of NLRP3 by MCC950 can suppress MUC5AC^+^ goblet cell differentiation and mucus production in pHBE cells from asthmatic patients. Similarly, MCC950 inhibits IL‐13‐induced goblet cell hyperplasia and mucus overproduction of pHBE cells from healthy donors in ALI cultures. Our results support an epithelial cell‐autonomous role for NLRP3 in the regulation of airway goblet cell differentiation and mucus production in asthma.

Recently, two potassium channels *Kcnk6* (encoding TWIK2) and *Kcnk13* (encoding THIK‐1) have been reported to trigger NLRP3 activation in macrophages [32, 33, 34]. Here, we identify KCNJ2 as the first potassium channel to promote NLRP3 activation in epithelial cells, thereby contributing to goblet cell hyperplasia and mucus overproduction in both asthmatic mouse models and asthmatic patients. Our findings also reveal a novel signaling axis governing goblet cell hyperplasia and mucus production through KCNJ2, NLRP3, SPDEF, and MUC5AC. *Kcnj2* inhibition in epithelial cells prevents aberrant activation of NLRP3 inflammasome, leading to reduced *Spdef* expression and subsequent downregulation of its downstream target gene *Muc5ac* in the lungs of OVA‐induced asthmatic mouse. Because *Kcnj2* inhibition also reduces IL‐13 levels induced by OVA in the lungs and IL‐13 can induce *Muc5ac* expression, it is possible that *Kcnj2* regulates *Muc5ac* expression by mediating both *Spdef* expression and IL‐13 production in asthmatic conditions. Importantly, pharmacological inhibition of KCNJ2 and/or NLRP3 similarly decreases expression of *SPDEF* and *MUC5AC* in ALI cultures of pHBE cells from asthmatic patients, suggesting a conserved role for the KCNJ2/NLRP3 axis in regulation of goblet cell differentiation and mucus production in the airway epithelium of asthmatic patients.

Potassium channels *Kcnk6* and *Kcnk13* drive NLRP3 activation by facilitating K^+^ efflux [32, 33, 34]. On the other hand, Ca^2+^ channels and intracellular Ca^2+^ levels are also crucial regulators of NLRP3 inflammasome activation and subsequent inflammatory responses [35]. Interestingly, our studies show that the potassium channel KCNJ2 links changes in intracellular Ca^2+^ levels and NLRP3 activation by regulating Ca^2+^ influx. Meanwhile, KCNJ2 can also regulate NLRP3 activation by mediating K^+^ efflux.

Recently, a study reports that reduced intracellular K^+^ concentration promotes a stable structural change in the inactive NLRP3, which induces an open conformation for the next step activation [36]. In our study, we find that KCNJ2 inhibition in airway epithelial cells results in reduced intracellular Ca^2+^ levels, increased intracellular K^+^ levels, and attenuated NLRP3 activation. These findings suggest that KCNJ2 may promote both Ca^2+^ influx and K^+^ efflux, thereby facilitating structural rearrangements and NLRP3 activation, as well as regulating its expression in airway epithelial cells in asthmatic conditions. It will be interesting to examine structure changes in NLRP3 in asthmatic airway epithelial cells after KCNJ2 overexpression or deletion. Furthermore, investigating whether KCNJ2 modulates signaling pathways known to regulate NLRP3 expression, such as NF‐κB signaling, may provide additional mechanistic insight.

*Kcnj2* deficiency in lung epithelial cells alleviates Th2 inflammation, reduces Th2 cytokine production, and decreases levels of lung epithelial cell‐derived alarmins, including IL‐33, TSLP, and CCL2, in both OVA‐ and HDM‐induced asthmatic mice, as well as in airway epithelial cells from asthmatic patients. NLRP3 deletion has been reported to inhibit production of IL‐33 and TSLP in mouse models of asthma [26]. It is thus possible that KCNJ2 promotes production of these alarmins by partially activating NLRP3 inflammasome in asthmatic conditions. Since many K^+^ channels are expressed in the respiratory epithelium [67], it is possible that multiple potassium channels coordinate to regulate NLRP3 activation within individual cells, or that distinct potassium channels function in different cell types to mediate NLRP3 activity. Such coordinated regulation may ultimately influence airway inflammation and airway remodeling in asthma.

To our knowledge, this study is the first to investigate the function of the susceptibility gene *Kcnj2* in asthma development. Our data demonstrates that *Kcnj2* plays a causal role in asthma development in an ion channel activity‐dependent manner. Given that potassium channels are druggable targets, *Kcnj2* may present a promising candidate for therapeutic intervention. Currently, ML133 and PA‐6 are two small‐molecular blockers of Kir2 channels [71]. It remains necessary to development more selective and potent blockers of KCNJ2 with minimal off‐target effects on other members of ion channels for future translational applications. Moreover, a genome‐wide association study (GWAS) in patients with chronic obstructive pulmonary disease (COPD) reveals that several SNPs in *KCNJ2* are also associated with bronchodilator responsiveness (BDR) [66]. It will be interesting to also explore the role of Kcnj2 in COPD development.

## 4 Experimental Section

### 4.1 Human Subjects

For the collection of lung tissue samples used for immunohistochemistry, asthmatic patients were recruited from subjects who underwent lung resection for a solitary pulmonary nodule. All biopsies were randomly obtained from the peripheral tissues at least 1 cm away from the nodule. Lung control samples were obtained from donors of lung transplantation in the First Affiliated Hospital of Guangzhou Medical University, Guangzhou, China. Due to the policy of data anonymization of healthy donors who underwent lung transplantation, only the clinical characteristics of asthmatic patients could be retrieved. Primary human bronchial epithelial (pHBE) cells from asthmatic patients and healthy controls were obtained by bronchoscopy with epithelial brushing. Informed written consents were obtained from all study participants. All asthma patients in the study were diagnosed according to the Global Initiative for Asthma (GINA) guidelines (https://ginasthma.org/).

### 4.2 Human Subject Approval

All human studies have been approved by the institutional review board at the First Affiliated Hospital of Guangzhou Medical University (ES‐2024‐K040‐01). Studies were carried out in compliance with the relevant ethical regulations.

### 4.3 Experimental Animals

The *Kcnj2^flox^* allele (C57BL/6J) was generated using the CRISPR‐Cas9 system and homology‐directed repair. The *Nkx2.1^Cre^* allele has been previously described [72]. *Kcnj2CKO* (*Nkx2.1^Cre^;Kcnj2^fl/f)^*) mice were generated by crossing *Kcnj2^fl/fl^* and *Nkx2.1^Cre^* mice. For genotyping of *Kcnj2* floxed mice, primers sets (loxP1‐Fwd: 5′‐TCTGTACCTGCCAATGTCAGCCTTG‐3′; LoxP1‐Rev: 5′‐ATGTACTTTAGATTGAATTGTGCGGA‐3′; and loxP2‐Fwd: 5′‐CAATTCTAGGAGAAACCAAGAAAGTTAG‐3′; LoxP2‐Rev: 5′‐GAATCACCGAGCTTGGAATGGGAGT‐3′) were used to generate ∼365 bp wild‐type and ∼489 bp loxP PCR amplicons for the 5'loxP site, and ∼240 bp wild‐type and ∼357 bp loxP PCR amplicons for the 3'loxP site. For genotyping of *Nkx2.1^Cre^* mice, primers sets (17400: 5′‐ CTCTGGTGGCTGCCTAAAAC‐3′; mIMR8744: 5′‐CAAATGTTGCTTGTCTGGTG‐3′; mIMR8745: 5′‐ GTCAGTCGAGTGCACAGTTT‐3′; mIMR9074: 5′‐ AGGCAAATTTTGGTGTACGG‐3′;) were used to generate ∼200 bp internal positive control and ∼300 bp transgene PCR amplicons. All mice were maintained under a 12‐h light and 12‐h dark cycle. All mouse husbandry was performed under standard conditions in accordance with institutional (Guangzhou Medical University) and national ethical and animal welfare guidelines. All protocols of animal experiments were approved by the Institutional Animal Care and Use Committees of Guangzhou Medical University (No.20240284). The use of animals in these experiments was in accordance with the guidelines issued by the Chinese Council on Animal Care.

### 4.4 OVA‐Induced Asthma Model

Eight‐week‐old female mice were used in the experiments. The experimental procedures for generating the mouse model of allergic asthma were as follows. Mice were performed intraperitoneal injection of 5% OVA dissolved in 0.9% saline (100 µL) and sensitization with Imject Alum (100 µL) (Invitrogen, 77161, Carlsbad, USA) on day 0 and day 7. Mice were then challenged with 5% OVA from day 14 to day 28 using an ultrasonic nebulizer for 30 min each day. Airway hyperresponsiveness (AHR) was examined 24 h after the last nebulization, followed by euthanasia of mice and sample collection for analysis.

### 4.5 HDM‐Induced Asthma Model and ML133 Treatment

Eight‐week‐old female mice were used in the experiments. Mice were sensitized intranasally with 10 µg of house dust mite (HDM) (Greer labs, RMB84M, Lenoir, USA) extract dissolved in saline from day 0 to day 3 (four times in total). From day 8 to day 14, mice were challenged with 10 µg of HDM intranasally once daily (seven times in total). Mice were intranasally administered 40 µL of ML133 at a concentration of 25 µM every three days from day 0 to day 12 (five times in total). Each mouse received a total intranasal solution volume of 40 µL per administration. AHR was measured on day 15, 24 h after the last HDM challenge. Mice were euthanized on day 16 for sample collection and analysis.

### 4.6 HDM‐Induced Asthma Model and Adeno‐Associated Virus Serotype 6 (AAV6)‐Mediated *Kcnj2* Overexpression

Eight‐week‐old female mice were used in the experiments. Mice were sensitized intranasally with 10 µg of HDM extract dissolved in saline from day 0 to day 3 (four times in total). From day 8 to day 14, mice were challenged with 10 µg of HDM intranasally once daily (seven times in total). On day 5, all mice received an intratracheal injection of AAV6‐Vector or AAV6‐*Kcnj2* overexpression virus (5 × 10^11^ genome copies (GC) per mouse in a total volume of 50 µL, sourced from Guangzhou PackGene Biotechnology Co., Ltd.). AHR was measured on day 15, 24 h after the last HDM or saline challenge. Mice were euthanized on day 16 for sample collection and analysis.

### 4.7 16HBE Cell Culture, HDM, and Chemical Treatment

16HBE cells were cultured in RPMI 1640 medium (C11875500CP, Gibco, Carlsbad, USA) containing 10% fetal bovine serum (100991‐141C, Gibco, Carlsbad, USA). 16HBE cells were treated with 100 µg/mL HDM extract, 30 µM BAPTA‐AM (S7534, Selleck, Houston, Texas, USA), 10 µM Ionomycin (HY‐13434, MedChemExpress, St Louis, MO, USA), and 30 µM ML133 (M2181, AbMole, Houston, Texas, USA) at the indicated time points, followed by sample collection for data analysis.

### 4.8 Fluo‐4 AM Staining in 16HBE Cells

16HBE cells were cultured in a 12‐well plate and washed twice with calcium‐free PBS before Fluo‐4 AM staining. 300 µL of 2.5 µM Fluo‐4 AM (Thermo Fisher Scientific, USA, #F14201) solution was added to incubate with cells per well at 37°C in a 5% CO_2_ incubator for 20 min. Cells were then washed with serum‐free medium without phenol red, followed by incubation with 500 µL of PBS per well at 37°C in a 5% CO_2_ incubator for 15 min. 500 µL of phenol red‐free medium was then added to each well, followed by imaging using a fluorescence microscope.

### 4.9 IPG‐4 AM Staining in 16HBE Cells

16HBE cells were cultured in a 12‐well plate for 24 h, and were then treated with 100 µg/mL HDM extract (Greer Labs, RMB84M, Lenoir, USA) and 30 µM ML133 for 23 h. Subsequently, 10 µM nigericin (Sigma‐Aldrich, USA, #N7143‐5, prepared as a 2.5 mM stock in ethanol) was added to inoculate with cells at 37°C for 30 min. Cells were then used for IPG‐4 AM staining (ION Biosciences, USA, #3021F). All reagents were pre‐warmed to room temperature. IPG‐4 AM was reconstituted by adding 25 µL DMSO, vortexed, and briefly centrifuged. The dye loading solution was prepared by mixing the IPG‐4 AM/DMSO solution with 9.9 mL HEPES buffer and 100 µL 100X Pluronic F‐127. After removing the culture medium, cells were washed twice with calcium‐free PBS. The 500 µL of dye loading solution was then added to incubate with cells per well at 37°C in a 5% CO_2_ incubator for 45 min. The dye solution was then replaced with 500 µL phenol red‐free medium for fluorescence imaging. IPG‐4 signals were normalized to untreated cells in the medium.

### 4.10 Air–Liquid Interface (ALI) Culture of pHBE Cells

ALI culture of pHBE cells was performed as previously described in our studies [41]. Briefly, after expansion of pHBE cells in culture medium (STEMCELL, # 05008) at 37°C in a 5% CO_2_ incubator, pHBE cells were seeded on membrane supports of 12‐well Transwell inserts (Corning, #3460). Each well received 1 mL and 0.5 mL of expansion culture medium in the basal compartment and the apical compartment, respectively, and cultured at 37°C in a 5% CO_2_ incubator. Once pHBE cells reached complete confluence, the culture medium was carefully aspirated from both compartments. The basal compartment was replenished with PneumaCult‐ALI Medium (STEMCELL, #05001) to initiate cell differentiation. ALI cultures were maintained under air‐liquid interface conditions by changing the medium in the basal compartment every 2 days. Starting from the second week, the cells were washed with D‐PBS (without Ca^2+^ and Mg^2+^) to remove excess mucus from the surface of the apical compartment. ALI cultures were treated with IL‐13, KCNJ2 inhibitors, NLRP3 inhibitor and NLRP3 agonists at the indicated time. Samples were collected for analysis on day 21. Immunostaining was performed using the following primary antibodies: Mouse anti‐MUC5AC (1:400, Thermo Fisher Scientific, MA5‐12178); Rabbit anti‐Acetyl‐α‐Tubulin (1:500, Cell Signaling Technology, #5335S), and Rabbit anti‐Ki67 (1:400, Thermo Fisher Scientific, PA5‐19462).

### 4.11 Human Airway Organoid Culture

pHBE cells were expanded in PneumaCult‐Ex Plus Medium (STEMCELL Technologies, USA, #05040) at 37°C in a 5% CO_2_ incubator. Following the manufacturer's instructions, Matrigel‐embedded airway organoids were generated using the PneumaCult Airway Organoid Kit (STEMCELL Technologies, USA, #05060). In brief, pHBE cells were seeded into Matrigel domes and cultured in PneumaCult Airway Organoid Seeding Medium for 7 days. Cell differentiation was initiated by switching to PneumaCult Airway Organoid Differentiation Medium and maintained for 28 days. Organoids were then harvested by removing the Matrigel using Gentle Cell Dissociation Reagent (Corning, USA, #354253) and incubated on a shaker at 2–8°C for 1 h. Organoids were washed with PBS, fixed in 4% paraformaldehyde (PFA) at 4°C for 2 h, dehydrated in a gradient of 10% and 30% sucrose, embedded in OCT, and sectioned at 6 µm for immunostaining. Immunostaining was performed using the primary antibody Mouse anti‐MUC5AC (1:400, Thermo Fisher Scientific, MA5‐12178).

### 4.12 Measurements of Inflammatory Cells and Cytokines in Bronchoalveolar Lavage Fluid (BALF)

BALF collection was performed as previously described [73]. Briefly, the bronchus of the left lung lobe was ligated. 0.5 mL of saline was instilled into the right lungs and gently aspirated three times. BALF was centrifuged to collect the supernatant for analysis. Cells in BALF were resuspended in 0.2 mL of PBS after the addition of red blood cell lysis buffer for cell counting. Eosinophil and lymphocyte counts were determined by Wright and Giemsa staining.

### 4.13 Hematoxylin and Eosin (HE)

The left lung tissues were fixed in 4% PFA overnight at 4°C, mounted in OCT embedding compound, and sectioned at 8 µm. For routine histology, tissue sections were stained with hematoxylin and eosin (Solarbio, Beijing, China). The inflammation assessment in HE staining sections was semi‐quantitatively graded based on a previously published evaluation method [24]: 0, indicating absence of inflammatory cell infiltration; 1, representing minimal inflammatory cell infiltration surrounding the airways; 2, denoting a single layer of inflammatory cells encircling the airways; 3, indicating 2–4 circles of inflammatory cells surrounding the airways; 4, signifying more than 4 circles of inflammatory cells surrounding the airways. Airway wall thickness was determined by measuring the thickness between the bottom of the airway smooth muscle layer and the surface of the airway epithelium layer using Image Pro Plus 6.0 (Media Cybernetics, Bethesda, MD).

### 4.14 Penh Measurements (Non‐Invasive Approach)

In accordance with previous studies [74], airway reactivity was assessed using the Whole Body Plethysmography (WBP) system (Buxco, USA). Prior to measurements, the instrument's airtightness was verified. Mice were then positioned in the primary chamber of the free‐breathing plethysmograph (PLY4111; Buxco Electronics Inc.) and connected to a ventilator (Model 683; Harvard Apparatus, Holliston, MA, USA). Lung resistance in response to both PBS and indicated concentrations of methacholine (0, 3.125, 6.25, 12.5, and 25 mg/mL) (Sigma, A2251, Louis, USA) was quantified utilizing the instrument.

### 4.15 Immunohistochemistry (IHC)

Lung paraffin sections were deparaffinized and dehydrated. Antigen retrieval was performed for 5–10 min using sodium citrate buffer, followed by treatment with 3% H_2_O_2_ for 20 min. The sections were incubated in blocking solution (PBS/3% BSA) for 1 h at RT, incubated in the primary antibody overnight at 4°C, washed, incubated in secondary antibodies for 2 h at RT, washed, and then mounted for imaging. The following primary antibodies were used: Rabbit anti‐KCNJ2 (1:50, R&D Systems, MAB9548) for human lung tissue sections; Rabbit anti‐MUC5AC (1:200, Abcam, ab198294); Rabbit anti‐KCNJ2 (1:150, Thermo Fisher Scientific, PA5‐72498) for mouse lung tissue sections; Rabbit anti‐NLRP3 (1:1400, Thermo Fisher Scientific, PA5‐79740), and Rabbit anti‐caspase‐1 (1:3000, Proteintech, #22915‐1‐AP).

### 4.16 Immunostaining of Cryosections

Lung tissues were fixed in 4% PFA overnight at 4°C, incubated in 10% sucrose for 24 h at 4°C, and in 30% sucrose until the tissue sank, mounted in OCT embedding compound, and sectioned at 8 µm. To perform immunostaining, sections were fixed in 4% PFA for 10 min, followed by incubation in permeabilization solution (0.2% Triton X‐100/PBS) for 20 min at RT., incubated in blocking solution (0.1% Triton X‐100/PBS/3% BSA or M.O.M. Mouse IgG Blocking Reagent for the mouse primary antibody) for 1 h at RT, incubated in primary antibodies overnight at 4°C, washed, incubated in secondary antibodies for 2 h at RT, washed, and then mounted for imaging. The following primary antibodies were used: Mouse anti‐MUC5AC (1:400, Thermo Fisher Scientific, MA5‐12178), and Rabbit anti‐MUC5B (1:200, Novus Biologicals, NBP1‐92151).

### 4.17 Enzyme Linked Immunosorbent Assay (ELISA)

Mouse serum and BALF were collected for ELISA. ELISA for each protein was performed according to the manufacturer's instructions of the kit. The mouse OVA‐specific IgE ELISA kit was from Biolegend (439807, San Diego, USA). The mouse OVA‐specific IgG1 ELISA kit was from Cayman (500830, Michigan, USA). The mouse HDM‐specific IgE ELISA kit was from Biolegend (Chondrex, Inc., 3037, Woodinville, WA, USA). The mouse HDM‐specific IgG1 ELISA kit was from Cayman (Chondrex, Inc., 3034, Woodinville, WA, USA). ELISA kits for IL‐4, IL‐5, and IL‐13 were purchased from Thermo (#BMS613INST, #EMIL5ALPHA, #88‐7137‐22). The ELISA kit for IL‐1β was purchased from Lanpai (LP‐M035210, Shanghai, China). The ELISA kit for MUC5AC was purchased from Novus (NBP2‐76704, Centennial, CO, USA). The ELISA kit for MUC5B was purchased from Lanpai (#LP‐M04630, Shanghai, CN).

### 4.18 Western Blotting

Mouse lung tissues were lysed using RIPA buffer (P0013B, Beyotime Biotechnology, Shanghai, China), supplemented with protease inhibitors (P1005, Beyotime Biotechnology, Shanghai, China). Lysates were centrifuged at 12 000×*g* for 10 min, subjected to SDS‐PAGE, and transferred to nitrocellulose membranes. Membranes were probed with primary and horseradish peroxidase‐conjugated secondary antibodies (ProteinTech, Chicago, USA) and were developed using an enhanced chemiluminescent detection system (Millipore, Massachusetts, USA). The primary antibodies used were KCNJ2, Thermo, #PA5‐72498, 1:1000; CAPS1, Proteintech, #22915‐1‐AP, 1:1000; MUC5AC, Thermo, #MA5‐12178, 1:1000; NLRP3, CST, #15101S, 1:1000; IL‐1β, Cell Signaling Technology, #12242S, 1:1000; SPDEF, Abmart, #PU149664S, 1:1000; Phospho‐NLRP3 (Ser194), Affinity Biosciences, # AF3555, 1:500; ACTB, Fdbio, #FD0060‐50, 1:5000; GAPDH, Proteintech, #60004‐1‐Ig, 1:10000.

### 4.19 Quantification of Western Blot Signals

The KCNJ2, MUC5AC, NLRP3, caspase‐1, IL‐1β, SPDEF, and ACTB levels were quantified using Image J. KCNJ2, MUC5AC, NLRP3, caspase‐1, IL‐1β, SPDEF, and ACTB levels were normalized to the values yielded by ACTB (Table 1).

**TABLE 1:** List of Primers.

Mouse Primers		
	Forward	Reverse
*mActb*	5'‐GGCTGTATTCCCCTCCATCG‐3'	5'‐CCAGTTGGTAACAATGCCATGT‐3'
*mKcnj2*	5'‐ATGGGCAGTGTGAGAACCAA‐3'	5'‐TCATATCTCCGATTCTCGCC‐3'
*mMuc5ac*	5'‐CAGGACTCTCTGAAATCGTACCA‐3'	5'‐GAAGGCTCGTACCACAGGG‐3'
*mSpdef*	5'‐GACGGACGACTCTTCTGACA‐3'	5'‐CTGTTCGTGGTGCCACATCT‐3'
*mFoxa3*	5'‐ATGCTGGGCTCAGTGAAGATGGA‐3'	5'‐AAGGTCATGTAGGAGTTGAGAGGG‐3'
*mCcl2*	5'‐GCTCAGCCAGATGCAGTTAAC‐3'	5'‐GCACAGACCTCTCTCTTGAGC‐3'
*mIl‐33*	5'‐GGGTACCAAGCATGAAGAGAAC‐3'	5'‐CAGGGAGGCAGGAGACTG‐3'
*mTslp*	5'‐GAAAGGGGCTAAGTTCGAGC‐3'	5'‐TAGCCTGGGCAGTGGTCA‐3'

Human Primers		
	Forward	Reverse
*hACTB*	5'‐CATCGAGCACGGCATCGTCA‐3'	5'‐TAGCACAGCCTGGATAGCAAC‐3'
*hIL‐33*	5'‐GTGACGGTGTTGATGGTAAGAT‐3'	5'‐AGCTCCACAGAGTGTTCCTTG‐3'
*hTSLP*	5'‐ATGTTCGCCATGAAAACTAAGGC‐3'	5'‐GCGACGCCACAATCCTTGTA‐3'
*hCCL2*	5'‐CAGCCAGATGCAATCAATGCC‐3'	5'‐TGGAATCCTGAACCCACTTCT‐3'
*hSPDEF*	5'‐TTCACTACTGTGCCTCGAC‐3'	5'‐AACTCCTTGAGGAACTGCC‐3'
*hMUC5AC*	5'‐ CAGCACAACCCCTGTTTCAAA‐3'	5'‐ GCGCACAGAGGATGACAGT‐3'

### 4.20 RNA Isolation and Quantitative PCR (RT‐qPCR)

Total RNA extraction was conducted using the Trizol extraction reagent (Invitrogen, SanDiego, CA). cDNA was synthesized using the reverse transcription reagent (Vazyme, Nanjing, China) according to the manufacturer's instructions. Quantitative real‐time PCR was performed using a CFX instrument (BioRad, Hercules, CA). *Actb* was used as the internal control. The following primers were used:

### 4.21 Statistical Analysis

Statistical analyses were performed using GraphPad Prism software (version 10.1). Data were presented as mean ± s.d. Sample sizes (n) were indicated in the figure legends. Data were normalized to the control group mean where applicable. Outliers were evaluated using the ROUT method (Q = 1%), and no data points were excluded unless otherwise stated. In cases where two groups were analyzed, samples were tested with unpaired two‐tailed Student's t‐tests. The Pearson correlation coefficient was used for the association analysis between the two groups. Penh measurements were analyzed using two‐way ANOVA followed by Tukey's post‐hoc test. In cases where three or more than three groups were analyzed, the analysis was performed using One‐way ANOVA followed by Tukey's post‐hoc test for multiple comparisons. *p < 0.05* indicates a finding is significant. *p ≥ 0.05* indicates a finding is not significant.

## Author Contributions

W.Y. conceived the project and designed experiments; Y.C. and S.W. contributed to experiments and data analysis; Y.P. recruited patients and collected clinical information from patients; L.C. collected pHBE cells; S.C. collected human lung tissue samples; S.L. provided clinical guidance; W.Y. and Y.C. wrote the manuscript; W.Y., R.P., and S.L. provided resources and supervised the study. All authors commented on the manuscript.

## Conflicts of Interest

The authors declare no conflicts of interest.

## Supporting information




**Supporting File 1**: advs74792‐sup‐0001‐SuppMat.docx.

**Supporting File 2**: advs74792‐sup‐0002‐Data.zip.

## Data Availability

The authors declare that all data supporting the findings of this study are available within the article and its supplementary information files or from the corresponding author upon reasonable request.
