# Supplementary material for: KCNJ2 is Required for NLRP3 Inflammasome Activation That Drives Allergic Airway Inflammation and Remodeling
Source: Adv Sci (Weinh). 2026 Apr 10;13(29):e17666. doi: 10.1002/advs.202517666 (PMC13205818; doi:10.1002/advs.202517666)
Supplement: Supplementary file 1 — Supporting File 1: advs74792‐sup‐0001‐SuppMat.docx. [file ADVS-13-e17666-s002.docx]

Supporting Information

KCNJ2 is required for NLRP3 inflammasome activation that drives allergic airway inflammation and remodeling

Yachao Cui^1^, Shumei Wu^1^, Yang Peng^2^, Yiqi Liu^1^, Li Che^2^, Shiying Chen^2^, Feng Zhang^1^, Dajiang Qin^3^, Shiyue Li^2,^*, Pixin Ran^1,^* and Wenguang Yin^1^*


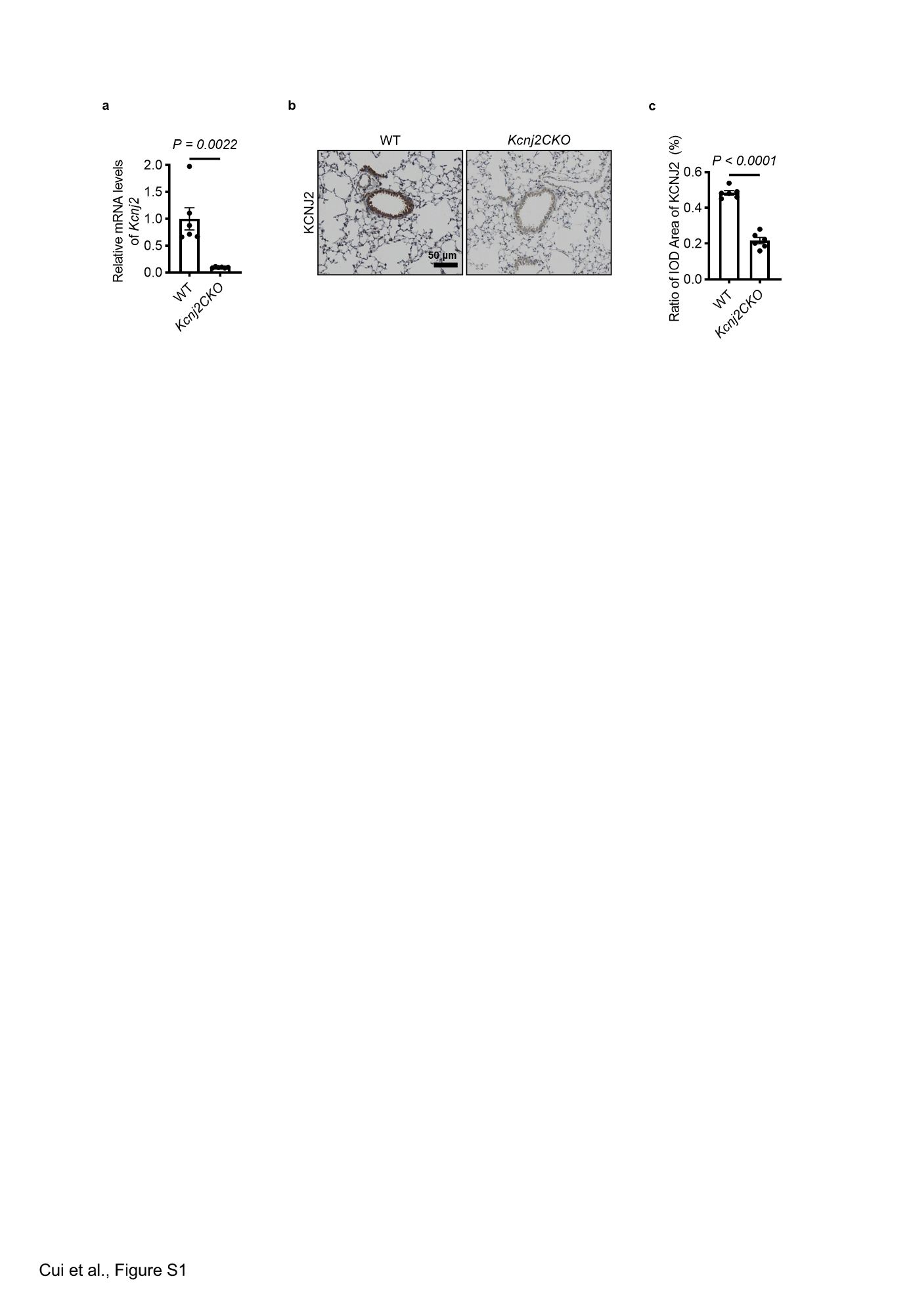


**Figure S1 KCNJ2 expression analysis in the lungs of WT and *Kcnj2CKO* mice.**

RT-qPCR analysis of *Kcnj2* in the lungs of WT (n = 6) and *Kcnj2CKO* (n = 6) mice. **b** Representative images of immunohistochemistry for KCNJ2 in the lungs of WT-saline (n = 6) and *Kcnj2*-*CKO* (n = 6) mice. **c** Quantification of average optical density values of KCNJ2 (as in **b**). Data are shown as mean ± s.d. Unpaired Students’ t-test.


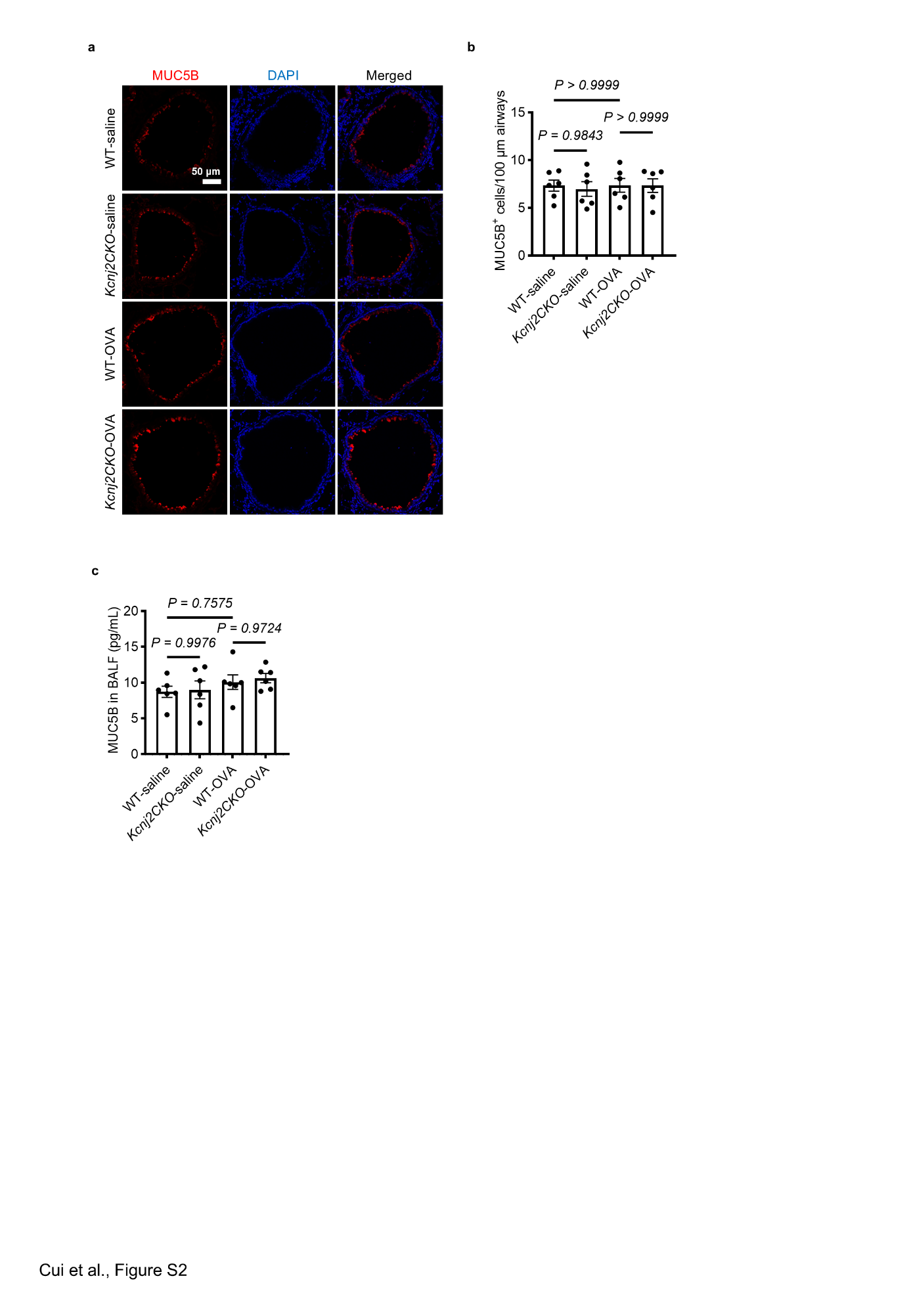


**Figure S2 *Kcnj2CKO* mice exhibit no obvious changes in the number of MUC5B^+^ cells in the airways or MUC5B protein levels in BALF compared with WT-OVA mice.**

**a** Immunostaining for MUC5B (red) and DAPI staining (blue) of lung sections of WT-saline (n = 6) and WT-OVA (n = 6) mice, and *Kcnj2CKO*-saline (n = 6) and *Kcnj2CKO*-OVA (n = 6) mice. **b** Quantification of MUC5B^+^ cells per 100-μm airway of WT-saline (n = 6) and WT-OVA (n = 6) mice, and *Kcnj2CKO*-saline (n = 6) and *Kcnj2CKO*-OVA (n = 6) mice. 4399, 4190, 4416 and 4400 MUC5B^+^ cells were analyzed for WT-saline (n = 6) and WT-OVA (n = 6) mice, and *Kcnj2CKO*-saline (n = 6) and *Kcnj2CKO*-OVA (n = 6) mice, respectively. **c** MUC5B protein levels in BALF of WT-saline (n = 6) and WT-OVA (n = 6) mice, and *Kcnj2CKO*-saline (n = 6) and *Kcnj2CKO*-OVA (n = 6) mice by ELISA. Data are shown mean ± s.d. one-way ANOVA.


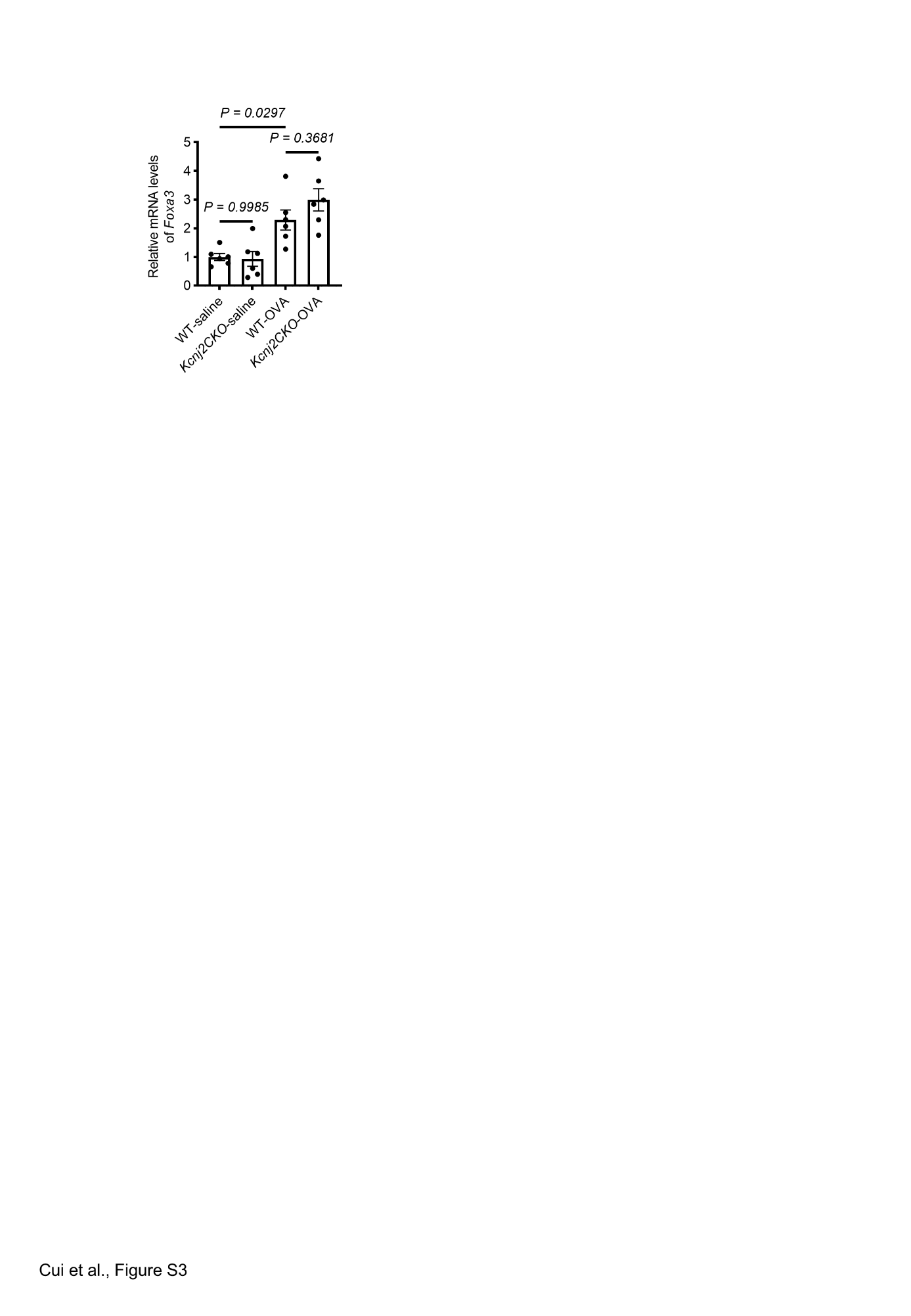


**Figure S3 *Kcnj2CKO*-OVA mice exhibit no obvious changes in *Foxa3* expression in the lungs compared with WT-OVA mice.**

RT-qPCR analysis of *Foxa3* in the lungs of WT-saline (n = 6) and WT-OVA (n = 6) mice, and *Kcnj2CKO*-saline (n = 6) and *Kcnj2CKO*-OVA (n = 6) mice. Data are shown as mean ± s.d. one-way ANOVA.


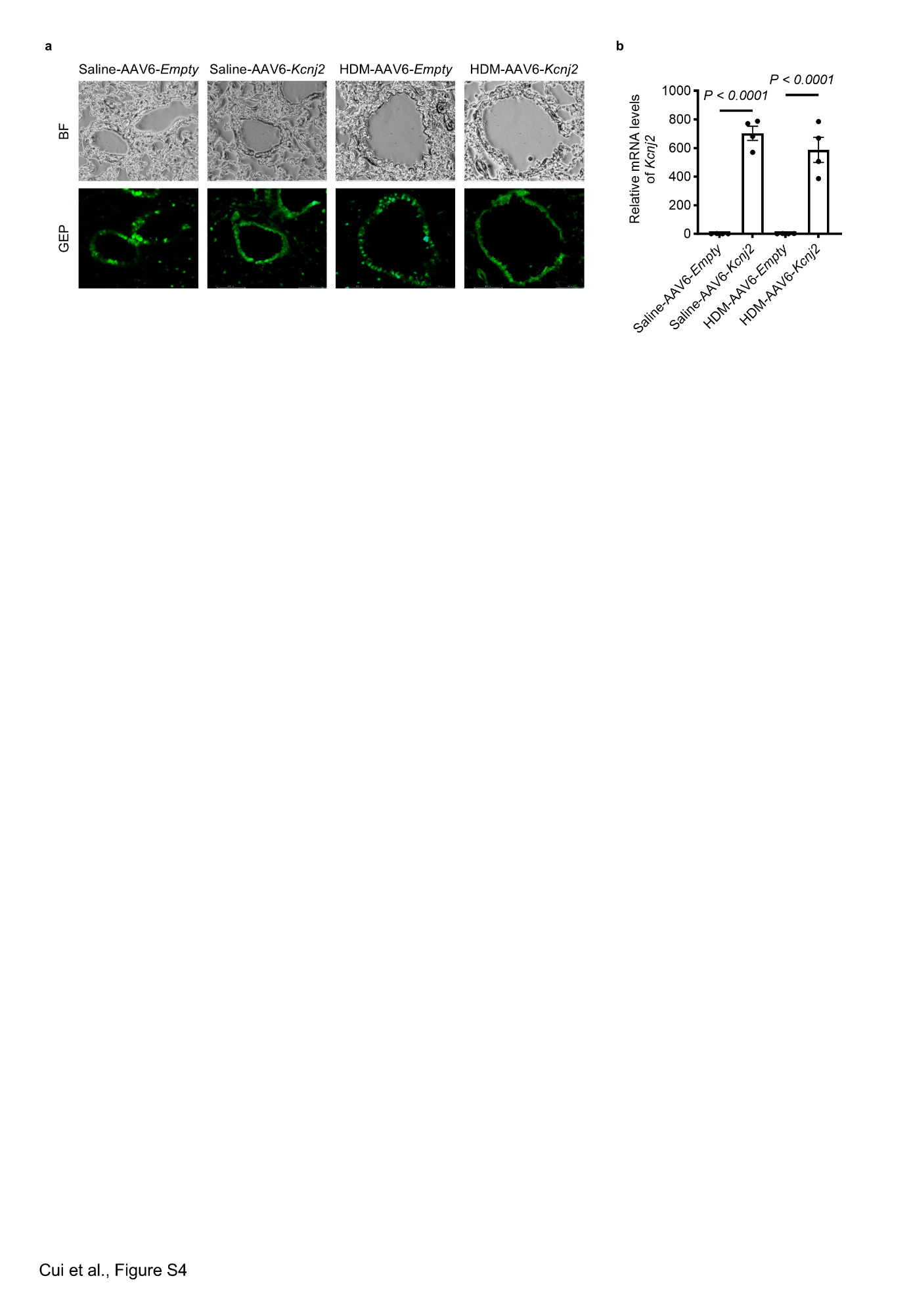


**Figure S4 KCNJ2 overexpression analysis in the lungs of HDM-induced asthmatic mice.**

**a** Representative fluorescence images of GFP expression in the lungs of saline-AAV6-Vector (n = 6), saline-AAV6-*Kcnj2* (n = 6), HDM-AAV6-Vector (n = 6), and HDM-AAV6-*Kcnj2* (n = 6) mice. **b** RT-qPCR analysis of *Kcnj2* in the lungs of saline-AAV6-Vector (n = 6), saline-AAV6-*Kcnj2* (n = 6), HDM-AAV6-Vector (n = 6), and HDM-AAV6-*Kcnj2* (n = 6) mice. Data are shown as mean ± s.d. one-way ANOVA.


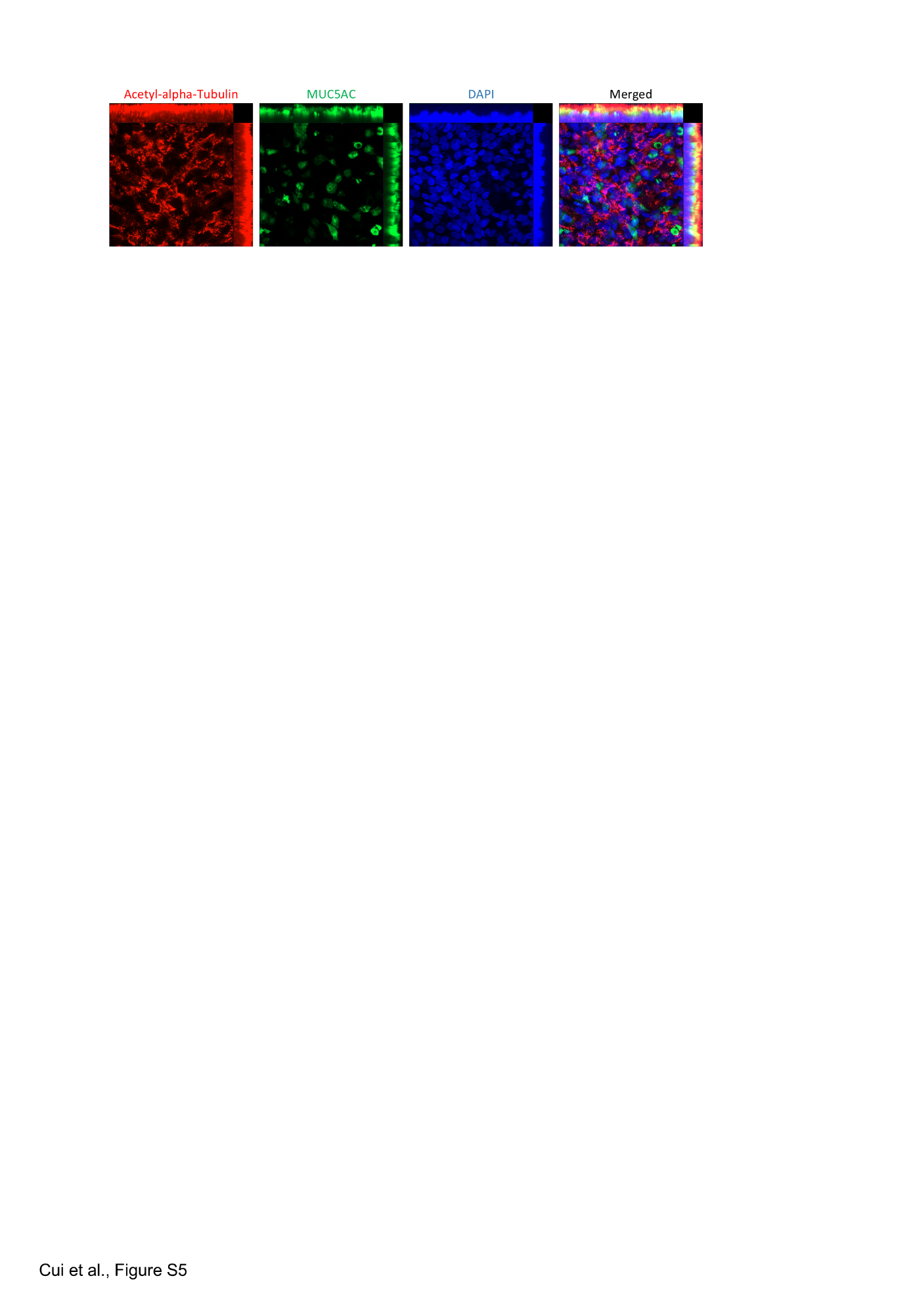


**Figure S5 Differentiation analysis of asthmatic pHBE cells at the air-liquid interface culture at day 21.**

Immunostaining for Acetyl-alpha-Tubulin (red) and MUC5AC (green), and DAPI staining (blue) in pHBE cells from asthmatic patients at the ALI at day 21. Representative confocal images demonstrate the presence of both ciliated cells and goblet cells.


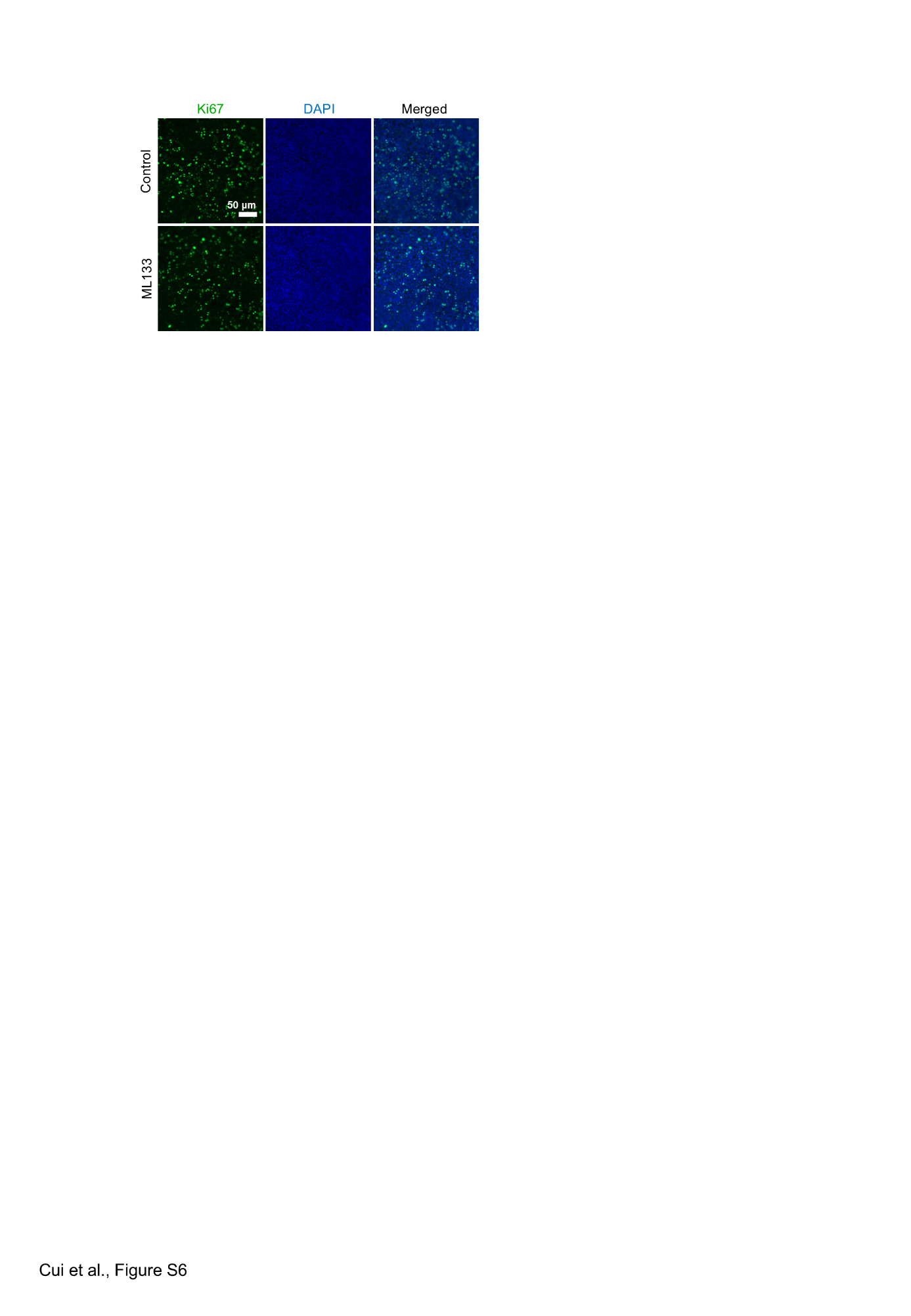


**Figure S6** **ML133 treatment causes no obvious differences in pHBE cell proliferation.**

Immunostaining for Ki67 (green) and DAPI staining (blue) in pHBE cells from asthmatic patients in ALI cultures after 21 days of DMSO or 30 μM ML133 treatment.


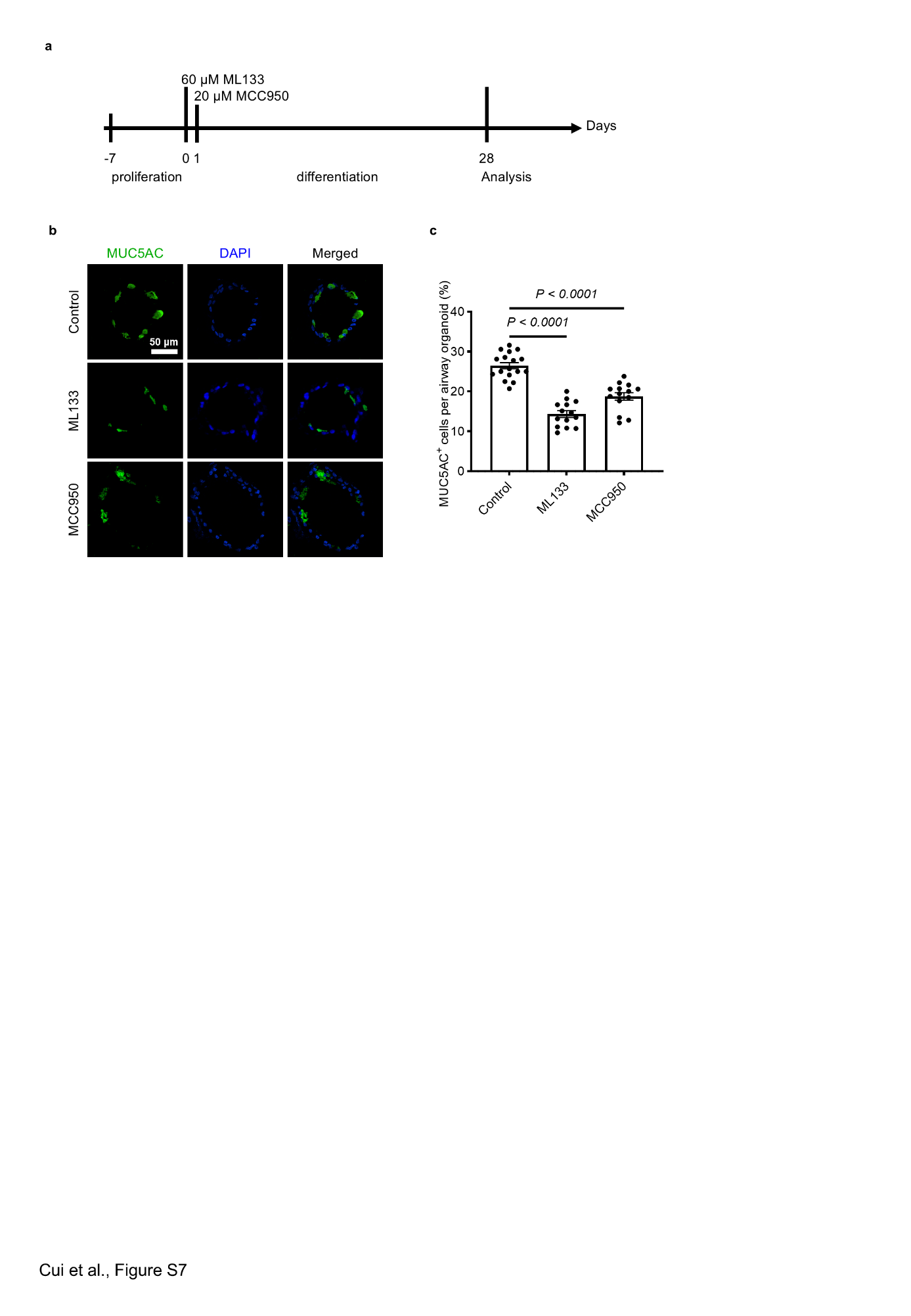


**Figure S7 KCNJ2 or NLRP3 inhibition reduces goblet cell differentiation in airway organoids of asthmatic patients.**

**a** Timeline for ML133 and MCC950 administration. **b** Immunostaining for MUC5AC (green) and DAPI staining (blue) in airway organoids of asthmatic patients after 28 days of DMSO or 60 μM ML133, or 27 days of 20 μM MCC950 treatment. **c** Percentage of MUC5AC^+^ goblet cells in airway organoids (as in **b**). 17, 14 and 14 organoids for DMSO, ML133 and MCC950 treatment groups were analyzed, respectively; 153, 68, and 89 MUC5AC^+^ goblet cells for each group were analyzed, respectively. Data are shown as mean ± s.d. one-way ANOVA.

**Table S1 Information of healthy donors and asthmatic patients for immunohistochemistry in Figure 1.**

| ID | Clinical Diagnosis | Gender  (M/F) | Age  (Year) | BMI  (Kg/m²) | FEV1  (L/min) | FEV1  chg% | FEV1/FVC  ratio |
| --- | --- | --- | --- | --- | --- | --- | --- |
| 1 | Asthma | F | 57 | 27.10 | 1.65 | -22.5 | 71.35 |
| 2 | Asthma | M | 56 | 27.26 | 2.22 | -26.09 | 65.79 |
| 3 | Asthma | F | 60 | 21.35 | 1.57 | -20.9 | 63.07 |
| 4 | Asthma | F | 45 | 23.63 | 1.69 | -26.83 | 60.82 |
| 5 | Asthma | F | 52 | 26.68 | 1.01 | -23.56 | 49.51 |
| 6 | Asthma | M | 54 | 24.02 | 2.84 | -17.29 | 64.52 |
| 7 | Asthma | M | 75 | 24.51 | 1.53 | -20.73 | 65.6 |

**Table S2 Information of healthy donors and asthmatic patients for pHBE cells at ALI cultures or in organoids in Figure 11, Figure 12, Figure S5, Figure S6 and Figure S7.**

| ID | Clinical Diagnosis | Gender  (M/F) | Age  (Year) | BMI  （Kg/m^2^） | FEV1  (L/min) | FEV1  chg% | FEV1/FVC  ratio |
| --- | --- | --- | --- | --- | --- | --- | --- |
| 8 | Asthma | F | 43 | 30.91 | 1.75 | -4.26 | 83.23 |
| 9 | Asthma | M | 58 | 21.70 | 1.73 | -22.76 | 68.17 |
| 10 | Asthma | F | 30 | 35.91 | 2.34 | -28.1 | 77.62 |
| 11 | Asthma | F | 51 | 23.67 | 2.34 | -24.8 | 74.61 |
| 12 | Healthy donor | M | 60 | not available | not available | not available | not available |
| 13 | Healthy donor | M | 52 | not available | not available | not available | not available |
| 14 | Healthy donor | F | 52 | not available | not available | not available | not available |
| 15 | Healthy donor | M | 60 | not available | not available | not available | not available |
| 16 | Healthy donor | F | 54 | not available | not available | not available | not available |
| 17 | Healthy donor | M | 56 | not available | not available | not available | not available |
